# Mechanistic insights into the calcium-mediated gelation of poly(4-*tert*-butylstyrene-*alt*-maleic acid)-*block*-poly(*N*-acryloylmorpholine) double hydrophilic block copolymers

**DOI:** 10.1016/j.eurpolymj.2026.114504

**Published:** 2026-02

**Authors:** Lauren E. Ball, Bennie Motloung, Michael-Phillip Smith, Rueben Pfukwa, Bert Klumperman

**Affiliations:** a Leibniz-Institut für Polymerforschung Dresden e.V., Hohe Straße 6, D-01069 Dresden, Germany; b Department of Chemistry and Polymer Science, Stellenbosch University, Private Bag X1, Stellenbosch 7599, South Africa

**Keywords:** Double hydrophilic block copolymer, Diblock copolymer, Calcium cross-linked, Hybrid polyionic complex, Block copolymer gels, Ionic strength, Z ratio, Injectable gel

## Abstract

Stimuli responsive double hydrophilic block copolymers (DHBCs) are ubiquitous in water-based applications, such as drug/gene delivery, nanoreactor and sensor development, photocatalysis, 3D inkjet printing, etc. DHBC-based gels, cross-linked with calcium ions, are particularly valuable for the development of biomedically relevant materials (such as wound dressings or injectable formulations), due to the benignity of Ca^2+^ in biological systems. Therefore, gels formed *via* the calcium-mediated crosslinking of poly(4-*tert*-butylstyrene-*alt*-maleic acid)-*block*-poly(*N*-acryloylmorpholine) (P*t*BuSMA-*b*-PNAM), which in itself constitutes polymers with established biomedical relevance, are promising candidates for the development of the aforementioned biomedical materials. To this end, P*t*BuSMA-*b*-PNAM diblock copolymers were synthesized with different block ratios (1:1, 1:2 and 2:1) and treated with Ca^2+^, whereby the concentration of the two constituents, the pH or the block copolymer architecture was varied, in order to tune the mechanical properties of the P*t*BuSMA-*b*-PNAM/Ca^2+^ gels. P*t*BuSMA-*b*-PNAM/Ca^2+^ with the lowest P*t*BuSMA composition (1:2 block ratio) could not form gels and rather formed micelles, whereas P*t*BuSMA-*b*-PNAM/Ca^2+^ with the highest P*t*BuSMA composition (2:1 block ratio) exhibited enhanced mechanical properties compared to the 1:1 block ratio. The overall amphiphilic balance of the P*t*BuSMA-*b*-PNAM/Ca^2+^ complexes was therefore proven vital for the design and formation of gels with desirable mechanical properties. P*t*BuSMA-*b*-PNAM/Ca^2+^ gels exhibited shear thinning when subjected to high shear conditions and demonstrated some self-healing properties, suggesting these materials have value in the formulation of injectable gels.

## 1 Introduction

Hydrogels are three-dimensional, hydrophilic polymeric networks, amenable to absorbing and retaining large amounts of water and/or biological fluids. They are especially attractive in biomedical applications, owing to their biomimetic nature, which allows for simulation of extracellular matrices and the natural cellular environment [1]. Despite their soft nature, owing to their usually high water content, the cross-linking within these materials permits the retention of remarkable mechanical properties. Conventional hydrogels (hereafter “gels”) require utilization of crosslinking agents in order to form permanent intermolecular covalent bonds [2]. However, physical crosslinking, which relies on secondary forces such as van der Waals forces, hydrogen bonding, hydrophobic and electrostatic interactions, has gained attention in recent times, largely due to advantages such as reversibility of gelation, tunable mechanical properties and stimuli-responsiveness [2,3]. Biomedicine has emerged as one area where the employment of gels has been prominent, particularly in wound dressings, tissue engineering and drug delivery systems [4].

Various polymeric materials, based on biopolymers or synthetic polymers, with variable architecture and composition can be utilized for the synthesis of gels. Alginate, for example, is a commonly employed biopolymer for this purpose, and is crosslinked through interaction of its carboxylate residues with metal ions (e.g. Ca^2+^, Fe^3+^, Al^3+^, Ba^2+^, etc.) [5]. Ca^2+^ in particular, is considered biologically benign and is vital for various biological functions, thus calcium crosslinked alginate gels are desirable materials for various medical applications [6–10]. Synthetic polymers afford the opportunity to synthesize innovative 3D networks as facile tuning of polymer chemical composition, topology, amphiphilicity, crosslink density and stimuli-responsiveness, for example, is possible [11]. Amphiphilic block copolymers are generally employed for the preparation of so-called physical hydrogels, but double hydrophilic block copolymers (DHBCs) have also garnered interest due to their wide use in water-based applications, including drug/gene delivery, utilization in nanoreactor or sensor development, photocatalysis and 3D inkjet printing [12,13]. DHBCs constitute hydrophilic blocks with diverse chemical composition, which may undergo self-assembly *via* the hydrophilic effect or *via* the application of a stimulus [12,14]. For non-stimuli responsive DHBCs, water is a non-selective solvent, thus self-assembly is promoted by a difference in water affinity between the blocks [15–18]. Contrarily, the hydrophobic effect drives self-assembly of amphiphilic block copolymers (i.e. water is a selective solvent for one block). Additionally, factors such as polymer architecture [19,20], polymer concentration [17,21] and block ratio [22] can be varied to afford diverse structures (e.g. micelles, vesicles, etc.) [23,24]. In contrast, amphiphilic characteristics can be imparted to responsive DHBCs through application of specific stimuli (i.e. pH, temperature, ionic strength, irradiation, complexation, redox reaction, etc.), reducing the aqueous solubility of one of the blocks and inducing self-assembly *via* the hydrophobic effect into various structures, including gels [14,25–30].

Various stimuli have been employed for the formation of DHBC-based gels, such as pH [31–35] and temperature [34–44], but most relevant to the present study is the addition of metal ions, which induces gel formation through DHBC-M^n+^ complexation. Nakagawa *et al*. reported calcium-crosslinked synthetic star DHBCs comprised of a dendritic polyester core and poly(oligo(ethylene glycol) methyl ether acrylate)-*b*-poly(acrylic acid) (DPE-*g*-POEGA-*b*-PAA) arms, which gelled rapidly under physiological conditions [45]. Unlike the star block copolymer, the related star random copolymers synthesized (DPE-*g*-POEGA-*r*-PAA) did not undergo gelation upon exposure to Ca^2+^, emphasizing the importance of the chemical and steric environment at the carboxylate-Ca^2+^ complexation sites. The DPE-*g*-POEGA-*b*-PAA/Ca^2+^ gels were probed with rheology, whereby the storage modulus (*G’*) was larger than the loss modulus (*G”*) and both moduli exhibited frequency independence across a wide range. This suggested that the DPE-*g*-POEGA-*b*-PAA/Ca^2+^ gels were behaving as viscoelastic solids. Additionally, the hydrogels possessed excellent biocompatibility *in vivo* and were shown to degrade within 5 h at 37 °C in culture media due to the loss of cross-linking points as Ca^2+^ was released from the gel. In a follow up study, Nakagawa *et al*. showed that DPE-*g*-POEGA-*b*-PAA/M^n+^ gels could be formed with a variety of metal ions (Zn^2+^, Cu^2+^, Al^3+^, and Fe^3+^) and demonstrated the efficacy of these materials for 3D inkjet printing [46]. The minimum concentration of metal ion required for gelation and the viscoelasticity of the formed gel varied depending on the stability of the metal-carboxylate complex, which was in turn influenced by the nature of the ionic species. In this regard, DPE-*g*-POEGA-*b*-PAA/Fe^3+^ yielded the highest resolution prints as it facilitated gelation at the lowest concentrations. Metal-ligand complexation has also been employed for the reversible-assembly of thermoresponsive microgels, for utilization in photocatalysis [47]. For example, poly[*N*-isopropylacrylamide-*co*-*N*,*N*’-methylene bis(acrylamide)]-*b*-poly(meth-acrylic acid) (P[NIPAm-*co*-MbA]-*b*-PMAA) microgels were synthesized and decorated with terpyridine functionalities *via* amidation [47]. The reversible assembly of the microgels was achieved *via* metal-terpyridine complexation (Ni^2+^, Fe^2+^, Co^2+^ or Zn^2+^), whereby weakly bound ions (Co^2+^, Zn^2+^) facilitated faster inter-particle exchange dynamics, yielding gels with much higher storage and loss moduli.

**Scheme 1:**
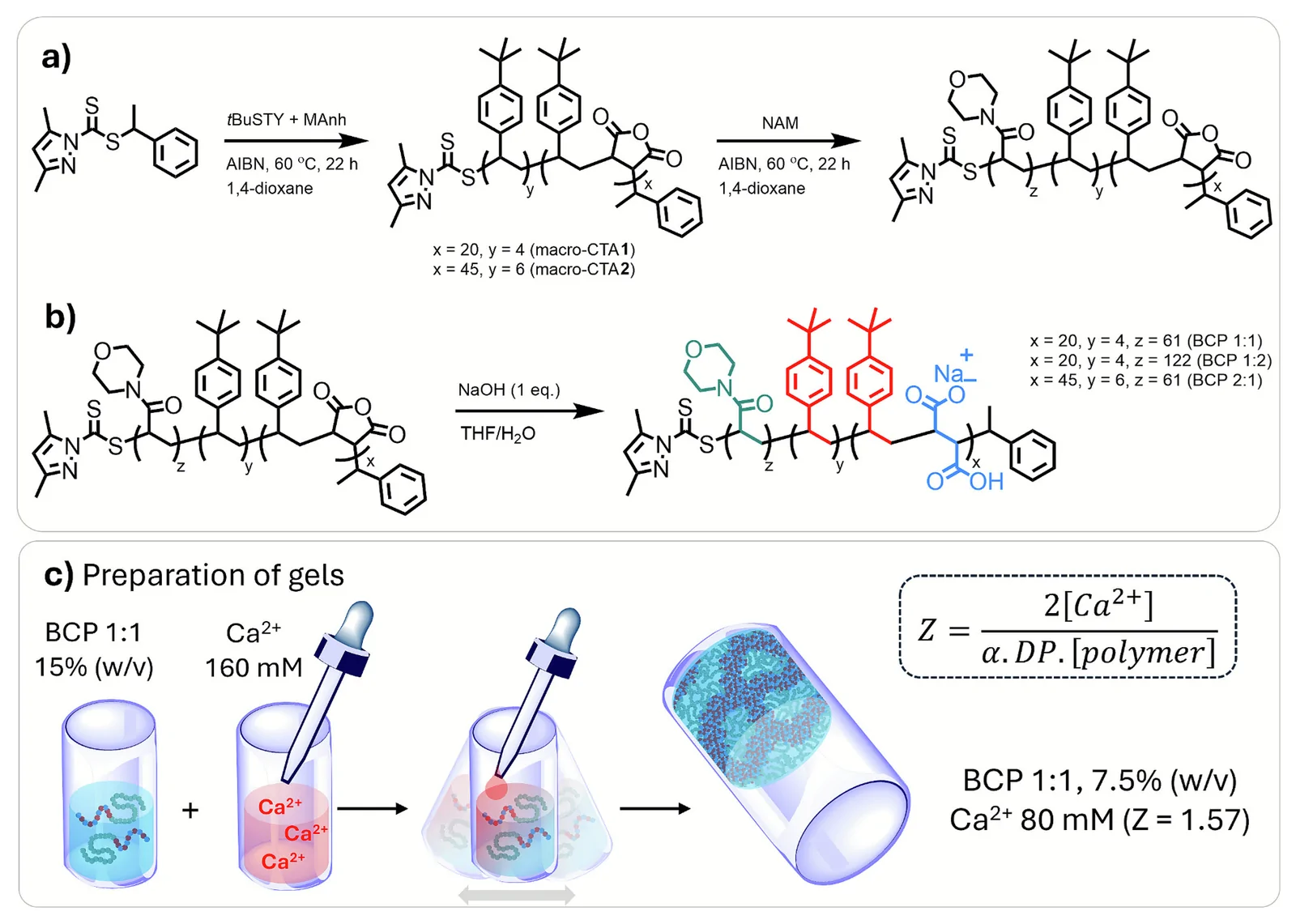
RAFT-mediated synthesis of P*t*BuSMAnh macro-CTAs and corresponding P*t*BuSMAnh-*b*-PNAM amphiphilic block copolymers **(a)**, followed by alkaline hydrolysis to yield P*t*BuSMA-*b*-PNAM DHBCs **(b)**. The preparation of gels is described in **(c)** and the Z ratio is defined, whereby α represents the charge of MA repeat units (e.g. α≈1 at pH 7.6), DP is the degree of polymerization and [polymer] is the concentration of the BCP.

To the best of our knowledge, reports of gels formed *via* the direct complexation of metal ions with hydrophilic linear diblock copolymers are limited. Generally, the interaction of M^n+^ with DHBCs, constituting a neutral (water-soluble) and ionic block, results in the formation of micellar or vesicular structures termed hybrid polyionic complexes (HPICs) [48]. HPIC formation using Ca^2+^ has been achieved using polymers such as poly(*N*-acryloylmorpholine)-*block*-poly(2-acryl-amidoglycolic acid) (PNAM-*b*-PAGA) [49], polyacrylate-*block*-poly(styrene sulfonate) (PA-*b*-PSS) [50], poly(2-(*N*-morpholino)ethyl methacrylate))-*block*-poly((methacryloyloxy)methyl phosphonic acid) (PMEMA-*b*-PMOMP) [51], poly(styrene-*alt*-maleic acid)-*block*-poly(*N*-acryloylmorpholine) (PSMA-*b*-PNAM) [52] and poly(4-*tert*-butylstyrene-*alt*-maleic acid)-*block*-poly(*N*-acryloylmorpholine) (P*t*BuSMA-*b*-PNAM) [52]. Complexation of Ca^2+^ to the carboxylate/phosphonate/sulfonate functional groups of these DHBCs decreases the charge density of the ionic block (according to the so-called Z ratio, defined in Scheme 1), forming hydrophobic domains which preclude interactions with water [48]. If an appropriate amphiphilic balance is obtained, *via* the formation of a significantly hydrophobic polyanion-Ca^2+^ phase, self-assembly into structures such as micelles is obtained.

The self-assembly of PSMA-*b*-PNAM/Ca^2+^ complexes into micelles (20–40 nm) was demonstrated by Klumperman and co-workers [52]. P*t*BuSMA-*b*-PNAM/Ca^2+^ complexes, which employed a polyanionic block with higher hydrophobicity and steric bulk around the chelation sites, did not afford micelles but rather a gel-like material. P*t*BuSMA-*b*-PNAM/Ca^2+^ complexes at varying DHBC and calcium content were probed using rheology, whereby stiffer gels could be obtained with increasing [DHBC] and [Ca^2+^]. Gel formation was proposed to occur *via* an interplay of chelation (between Ca^2+^ and carboxylate pendant groups), hydrogen bonding (between morpholine and carboxylic acid pendant groups) and hydrophobic interactions.

P*t*BuSMA-*b*-PNAM and polymers which constitute SMA-type co-polymers and PNAM, in general, have high biomedical relevance [52–56]. To assess whether P*t*BuSMA-*b*-PNAM/Ca^2+^ gels have potential in the formulation of biomedically relevant materials (such as injectable hydrogels), this study investigated the effect of varying structural (i.e. block ratio and architecture) and experimental parameters (i.e. polymer/Ca^2+^ concentration or pH) on gel formation and gel mechanical properties. P*t*BuSMA-*b*-PNAM with three different block ratios (1:1, 1:2 and 2:1) was synthesized *via* reversible addition-fragmentation chain transfer (RAFT) polymerization. Gels with varying composition were prepared according to procedures outlined in Scheme 1 and their rheological properties probed. A representative gel was subjected to high shear conditions followed by a thixotropy step test, to demonstrate that P*t*BuSMA-*b*-PNAM/Ca^2+^ gels undergo shear-thinning during injection and can subsequently recover their structure post-injection, respectively. This investigation would suggest that P*t*BuSMA-*b*-PNAM/Ca^2+^ complexes are indeed promising materials for biomedical applications.

## 2 Results and discussion

### 2.1 Synthesis of BCPs

**Fig. 1:**
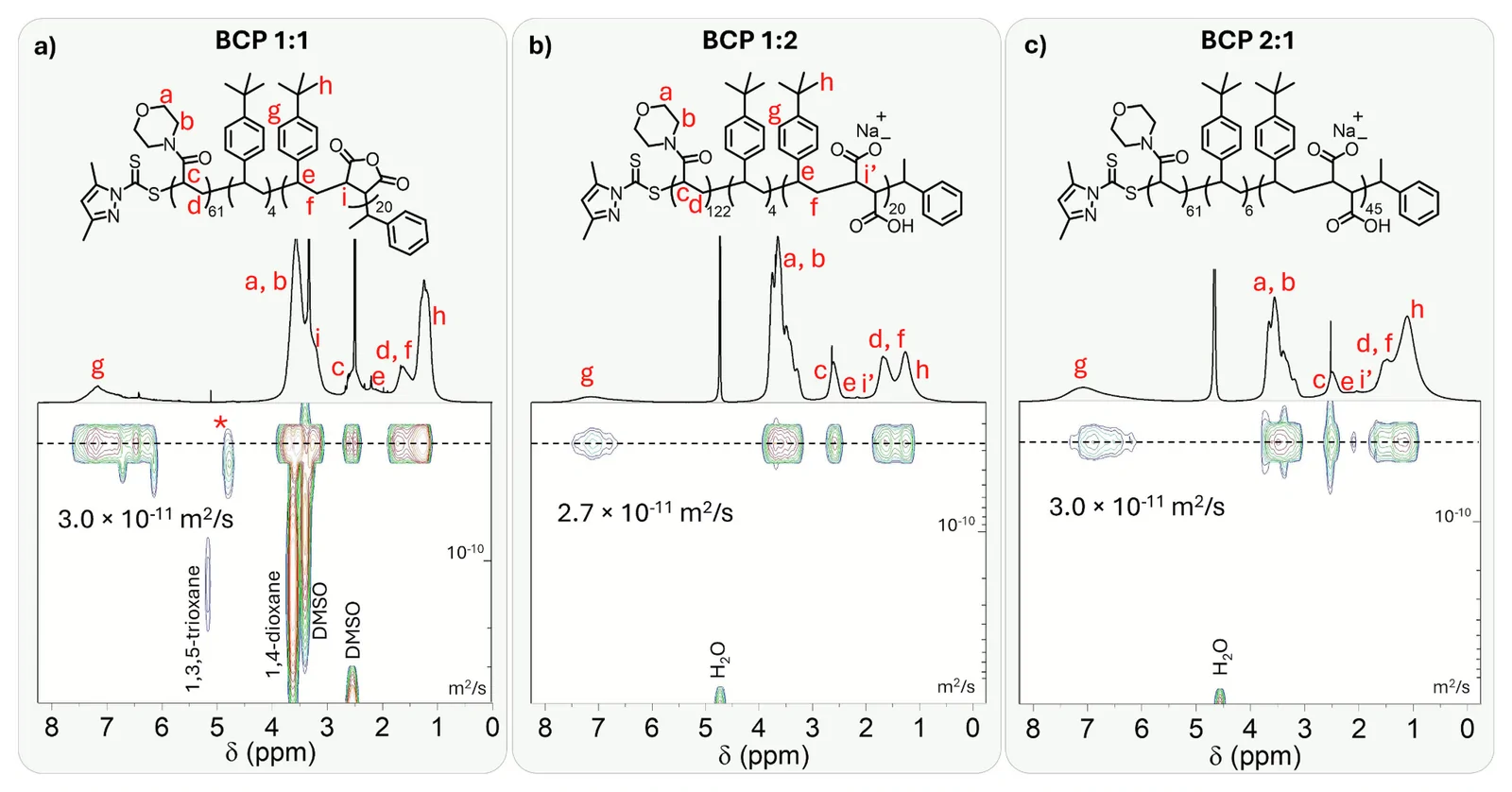
DOSY NMR spectroscopic analysis of (a) BCP 1:1 (before alkaline hydrolysis, in DMSO-*d*_*6*_), (b) BCP 1:2 (after alkaline hydrolysis, in D_2_O) and (c) BCP 2:1 (after alkaline hydrolysis, in D_2_O). Diffusion coefficients and residual solvent signals are indicated on each 2D spectrum. The signal marked with an asterisk in (a) corresponds to H_c_ of the NAM repeat unit connected to the thiocarbonylthio group.

All macro-CTAs and block copolymers were synthesized according to previously reported procedures [52]. P*t*BuSMAnh macro-CTAs with a DP of 44 (macro-CTA**1**, entry 1, Table 1) and 96 (macro-CTA**2**, entry 2, Table 1) were synthesized with near quantitative monomer conversion, yielding an alternating block of P*t*BuSMAnh with a short segment of 4–6 *t*BuSTY units at the ω-chain end (as confirmed *via* ^1^H NMR spectroscopy). The inclusion of the *t*BuSTY tail has been shown to improve the chain end fidelity of 3,5-dimethyl pyrazole-functional macro-CTAs [57]. SEC analysis showed that P*t*BuSMAnh with low *Ð* (1.24–1.32) had been successfully synthesized, with ${\text{M}}_{\text{n}}^{\text{SEC}}$ corresponding well with the calculated ${\text{M}}_{\text{n}}^{\text{theo}\;}$. Both macro-CTAs were utilized to mediate the RAFT polymerization of NAM, to produce P*t*BuSMAnh-*b*-PNAM block copolymers with three different block ratios (i.e. 1:1, 1:2 and 2:1, determined using ${\text{M}}_{\text{n}}^{\text{SEC}}$, Table 1). Despite a similar concentration of AIBN applied (2.1–2.7 mM) for the synthesis of BCP 1:1 and 1:2 (entry 3 & 4), the latter block copolymerization exhibited very low monomer conversion (7 %). In a study by Ball *et al*., the P*t*BuSMAnh-mediated polymerization of NAM displayed an initialization period (ca. 2 h) which was influenced by the nature of the macro-CTA [52]. It is possible that the lower concentration of macro-CTA**1** (13 mM) used for the synthesis of BCP 1:2 (entry 4) compared to the concentration applied during the synthesis of BCP 1:1 (20 mM) could have extended this initialization period. Therefore, the AIBN: macro-CTA**1**/**2** ratio and monomer concentration was increased (while maintaining similar monomer: macro-CTA**1**/**2** ratio) for the synthesis of BCP 2:1 (entry 6) and the repeated synthesis of BCP 1:2 (entry 5), resulting in quantitative NAM conversion (Table 1). While the SEC analysis for both BCP 1:1 and BCP 1:2 showed that low *Ð* (1.21) block copolymers had been synthesized, BCP 2:1 displayed a more significant shoulder at low elution volumes (corresponding to higher molecular weight polymer, Fig. S1) resulting in a higher *Ð* (1.52). This could be due to the higher concentration of AIBN employed, which increases the prevalence of termination or chain transfer side reactions [58]. In this instance, the latter is likely to be more prevalent as the UV eluograms (320 nm) exhibited a similar shoulder at low elution volumes (Fig. S1), suggesting the presence of thiocarbonylthio-functional polymers and not the products of termination by combination. Nevertheless, SEC analysis indicated that chain extension of the P*t*BuSMAnh macro-CTAs was successful as BCP molecular weight distributions had shifted to lower elution volumes than the corresponding macro-CTA (Fig. S1). Additionally, DOSY NMR analysis of each block copolymer showed that protons characteristic of the P*t*BuSMAnh block (H_g_, Fig. 1) and PNAM block (H_a_ and H_b_, Fig. 1) had similar diffusion coefficients, further suggesting that the block copolymerizations were successful [52].

**Fig. 2:**
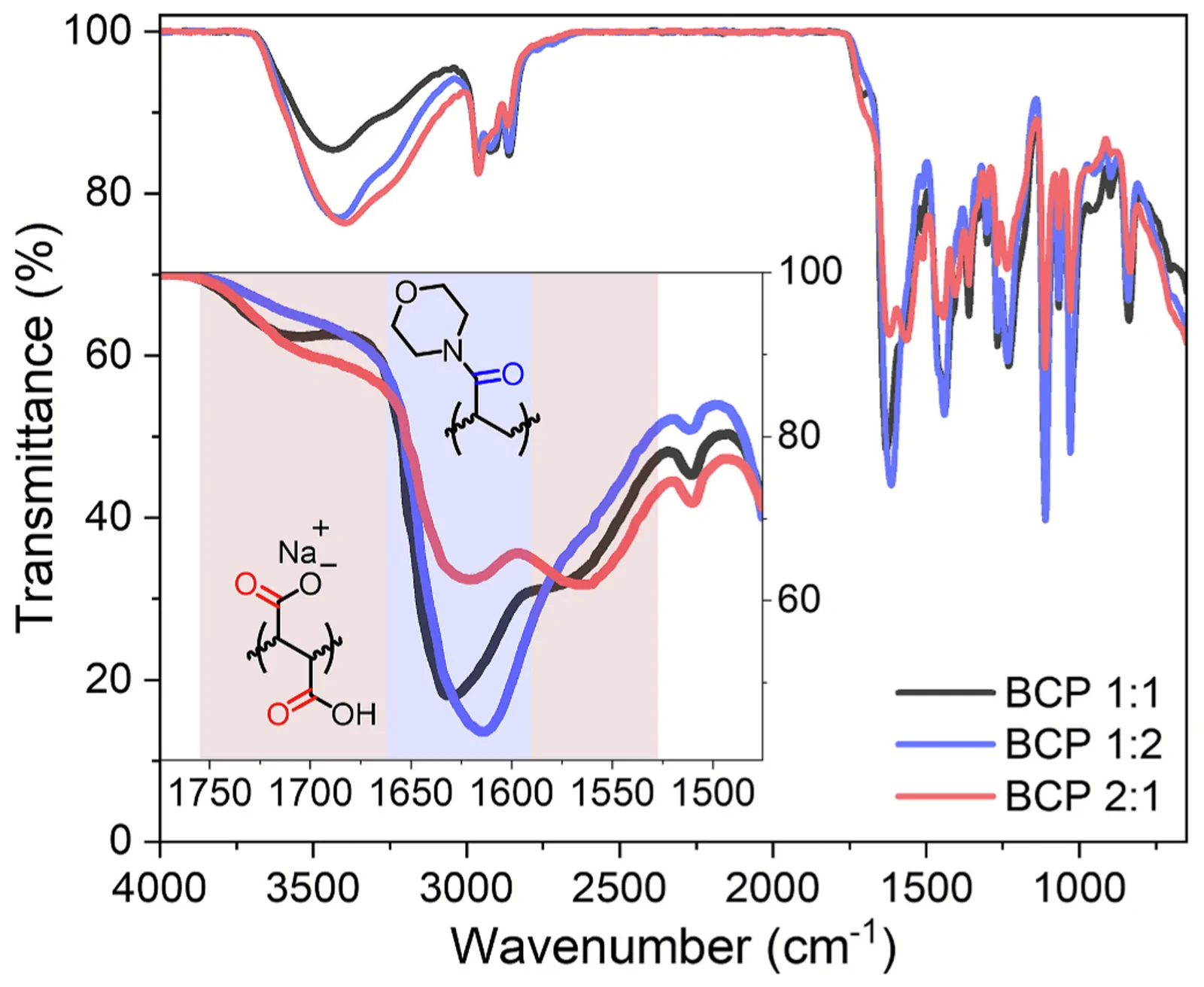
ATR-FTIR spectroscopic analysis of BCP 1:1, 1:2 and 2:1 after alkaline hydrolysis, dialysis and lyophilization. The inset data indicates the characteristic carbonyl stretching frequencies of PNAM (1615–1630 cm^−1^) and P*t*BuSMA carbonyl groups.

The amphiphilic P*t*BuSMAnh-*b*-PNAM block copolymers (entry 3, 5 & 6, Table 1) underwent alkaline hydrolysis to afford their DHBC derivatives (i.e. P*t*BuSMA-*b*-PNAM), using a previously reported protocol [52]. Upon exposure to the alkaline aqueous medium, MAnh repeat units underwent hydrolysis to afford maleic acid (MA) repeat units (confirmed *via* ATR-FTIR spectroscopy, Fig. 2). MAnh repeat units exhibit carbonyl stretching frequencies at approximately 1856 and 1775 cm^−1^, which were absent in the spectra presented in Fig. 2 [52]. Signals characteristic of carboxylic acid/carboxylate carbonyl stretching frequencies were observed at 1565–1710 cm^−1^, which would suggest the successful formation of MA repeat units [52].

### 2.2 Formation of BCP-based gels

In a previous study, we demonstrated for the first time that under appropriate conditions (i.e. in the presence of sufficient Ca^2+^), aqueous solutions of the P*t*BuSMA-*b*-PNAM block copolymer (with a 1:1 block ratio) could form a gel-like material [52]. The successful formation of these P*t*BuSMA-*b*-PNAM/Ca^2+^ gels (hereafter “BCP gels”) depended primarily on the BCP solid content, as well as the concentration of Ca^2+^ applied. Herein, through a systematic approach, we critically evaluate the exact conditions required for gel formation, as well as the proposed gelation mechanism facilitating the formation of these BCP-based gels.

**Fig. 3:**
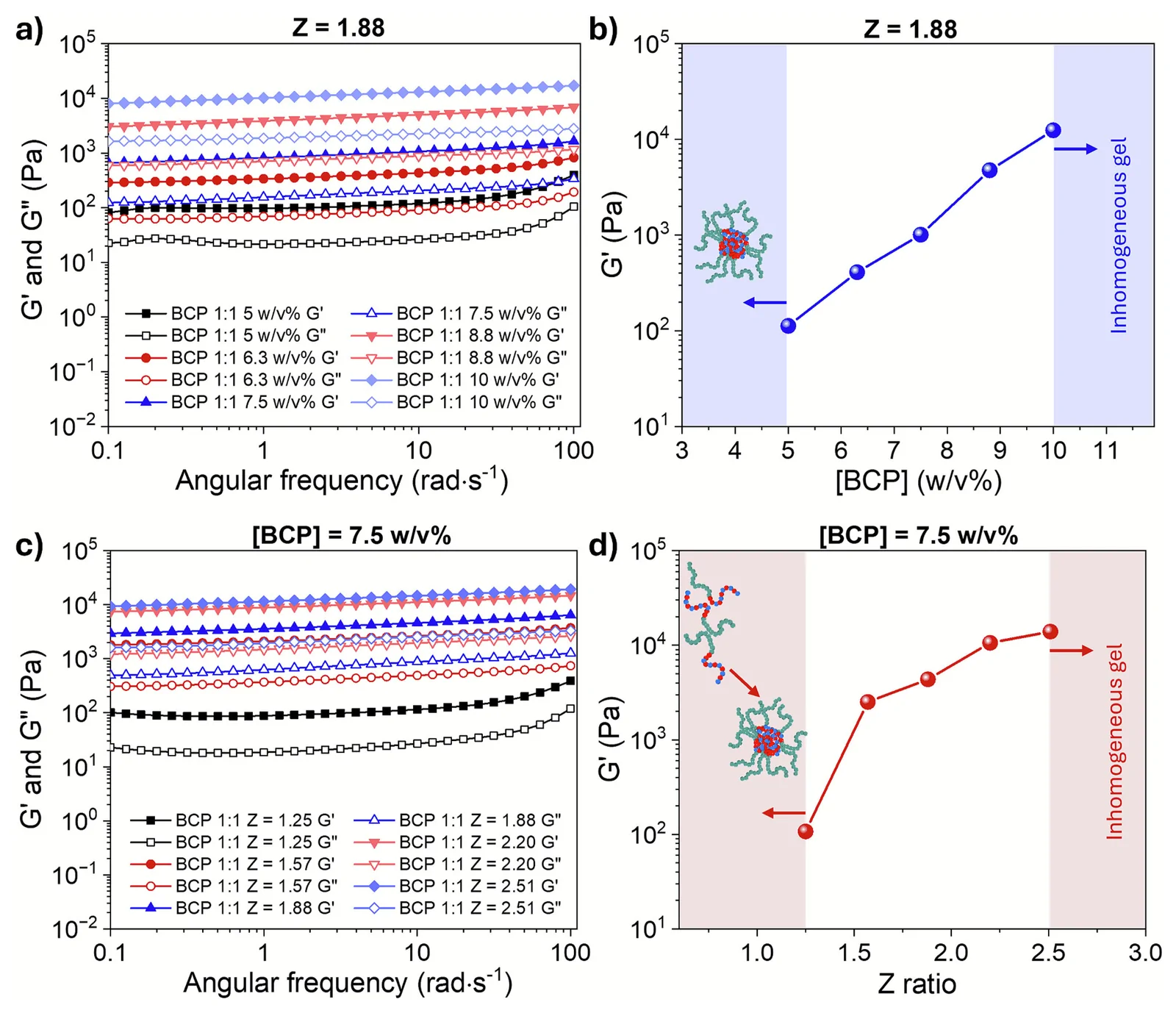
Frequency sweeps of P*t*BuSMA-*b*-PNAM BCP 1:1 gels (a) at different BCP concentrations (5–10 % w/v) and fixed Z ratio (1.88) and (c) at different Z ratios (1.25–2.51) and fixed BCP concentration (7.5 % w/v). Evolution of the elastic modulus of the gels as a function of (b) BCP concentration and (d) Z ratio, the data was extracted at 6.28 rad⋅s^−1^ from (a) and (c), respectively. Frequency sweep experiments were performed at a constant strain (1 %).

The first parameter to be investigated was the effect of solid content on the gel properties, therefore, gelation experiments were conducted at different BCP concentrations, ranging from 5 to 10 % (w/v) (in Tris HCl buffer, 50 mM, pH 7.6), while the Z ratio was fixed at 1.88 throughout the series. The utilization of BCP solid content > 10 % (w/v) promoted fast gelation kinetics and thus resulted in the formation of stiff and inhomogeneous gels (i.e. inconsistent opacity). The mechanical properties of the prepared BCP gels were probed *via* rheology, and the frequency sweep data is presented in Fig. 3. The frequency sweeps were performed at small strain amplitudes (i.e. 1 %), as determined by the linear viscoelastic (LVE) region measurements from amplitude sweep experiments. For all BCP gels assessed (Figs. 3a and c), the elastic modulus (*G’*) was significantly greater than the viscous modulus (*G”*) throughout the investigated angular frequency (hereafter “frequency”) range, suggesting a crosslinked material and predominantly elastic behavior (i.e. tan δ < 1) [59]. However, owing to their viscoelastic nature, the elasticity of the BCP gels depended on the frequency applied. The frequency dependence was more evident at 5.0 and 6.3 % (w/v), however, it was less pronounced in the 7.5–10 % (w/v) BCP concentration range, suggesting higher crosslink density and the formation of a stable gel-network (Fig. 3a) [60]. The stiffness of the gels was directly related to the BCP concentration, particularly at lower frequency range (Fig. 3b).

This could be ascribed to the availability of more block copolymer chains which facilitate the formation of a denser gel network [60]. A BCP solid content of 7.5 % (w/v) was the midway point between the onset of gelation and the formation of inhomogeneous gels and afforded materials with good homogeneity and visual consistency. Therefore, this BCP concentration was deemed optimal for subsequent experiments and used to prepare BCP gels at different Z ratios (i.e. 1.25–2.51) by varying the concentration of Ca^2+^ cations applied (Fig. 3c). Increasing the Z ratio gave rise to a corresponding increase in the stiffness of the BCP gels (Fig. 3d), owing to the availability of more Ca^2+^ cations, which increased the crosslink density between P*t*BuSMA blocks.

**Fig. 4:**
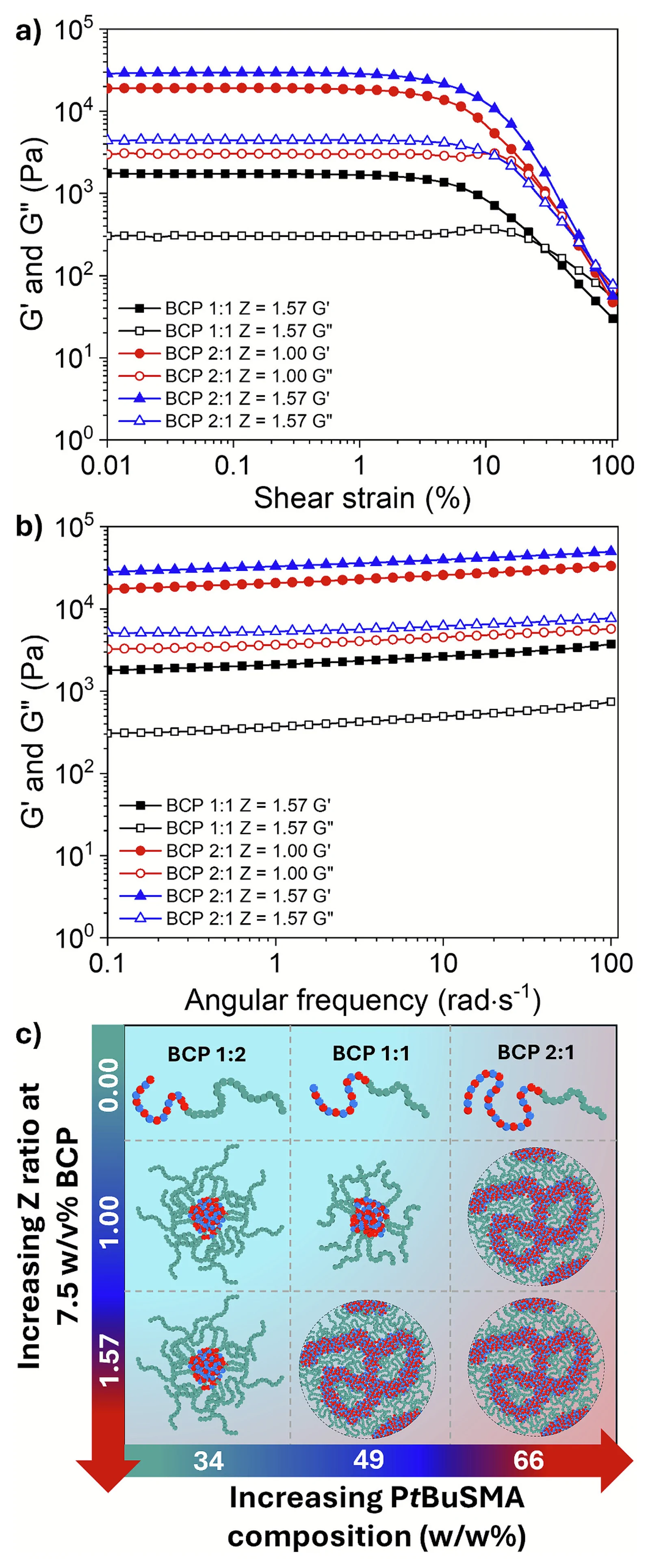
Amplitude **(a)** and frequency **(b)** sweeps of P*t*BuSMA-*b*-PNAM BCP gels at different block ratios (1:1 and 2:1), Z ratios (1.00 and 1.57) and a fixed BCP concentration (7.5 % (w/v)). Amplitude and frequency sweep experiments were performed at constant angular frequency (10 rad⋅s^−1^) and strain amplitude (1 %). The relative solution behavior of the different BCPs is summarized in **(c)**, as a function of Z ratio and the P*t*BuSMA composition (w/w%).

**Fig. 5:**
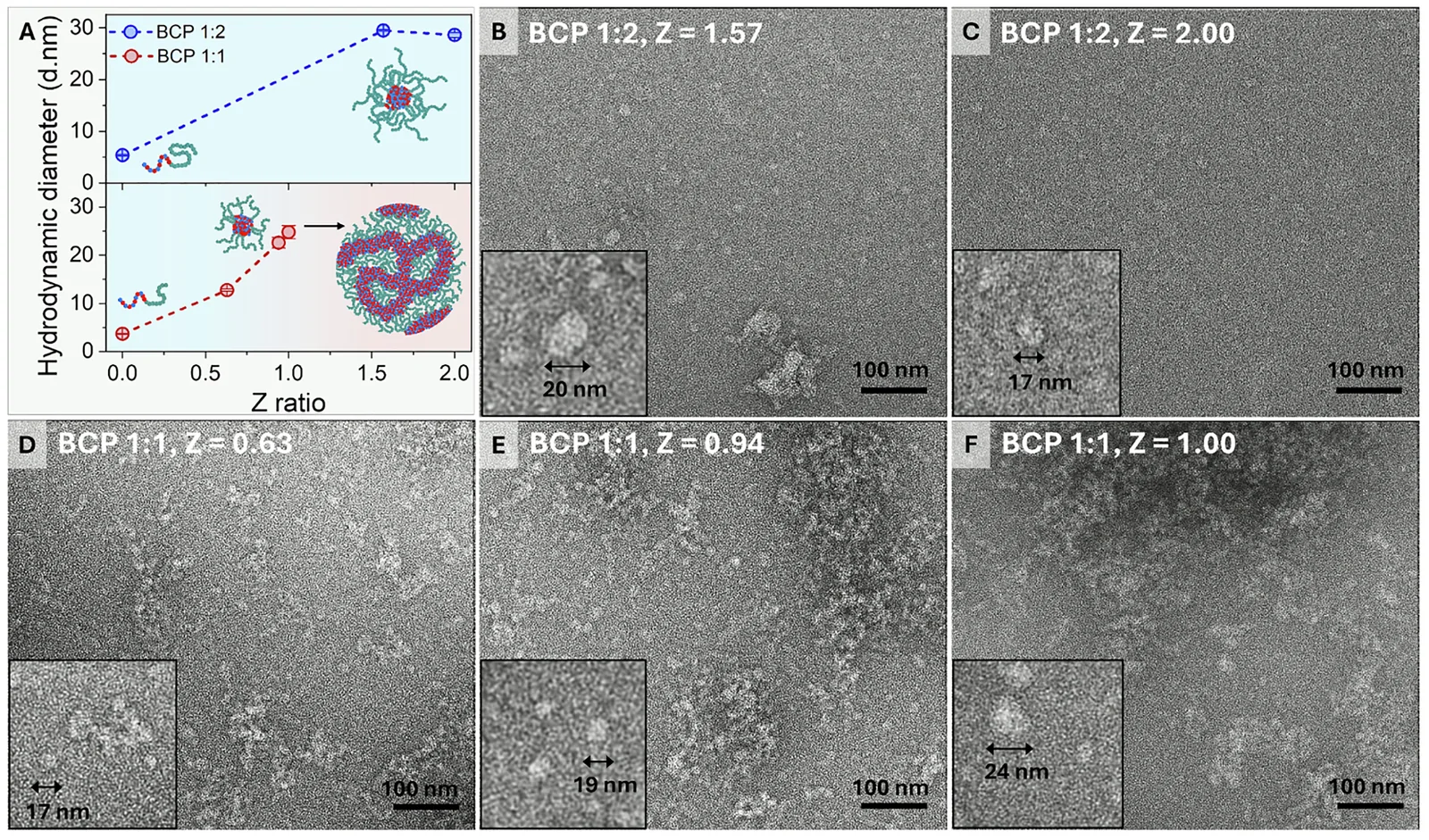
DLS measurements performed in triplicate for (A) for BCP 1:1 and 1:2 solutions prepared at 7.5% (w/v) and diluted to 0.5% (w/v) with the Z ratio varying between 0 and 2. These solutions were subsequently analysed *via* TEM (B–F).

So far, only BCP 1:1 has been used to form gels. To assess and compare the gelation of BCPs with different block ratios, ‘optimal’ gelation conditions for the BCP 1:1 gel were defined. The optimal conditions chosen were 7.5 % (w/v) BCP solid content and a Z ratio of 1.57, using the same considerations for homogeneity described *vide supra*. Subsequently, these conditions were applied to P*t*BuSMA-*b*-PNAM block copolymers with different block ratios (i.e. 1:1, 2:1 and 1:2), to assess the effect of varying content of the chelating P*t*BuSMA block (Figs. 4–5). Amplitude sweeps of the BCP 1:1 and 2:1 gels were performed at a constant angular frequency of 10 rad•s^−1^ and the amplitude of oscillation was varied between 0.01 % and 100 %. All the BCP gels assessed exhibited the behavior of viscoelastic solids (*G’* > *G”*), with both elastic and viscous moduli showing no dependence on the applied shear strain, particularly at lower shear strain regions (Fig. 4a). This plateau region represents the so-called LVE region, and in this regime, all the assessed gels displayed dominant elastic characteristics. The linearity limit was reached at shear strain values just below 10 %, as a result, the subsequent frequency sweep measurements were performed at 1 % shear strain to avoid permanent deformation during analysis. At Z ratio of 1.57, the BCP 2:1 gel displayed superior mechanical properties over the BCP 1:1 gel (Fig. 4b). Reducing the Z ratio of the BCP 2:1 gel to 1.00 still formed a stiffer gel than the BCP 1:1 gel at 1.57 as observed from a greater elastic modulus. At 7.5 % (w/v) solid content, the BCP 2:1 constitutes the highest proportion of the chelating P*t*BuSMA component, thus for a given Z ratio BCP 2:1 likely exhibits a higher crosslink density than BCP 1:1. This emphasizes the crucial role played by the chelating P*t*BuSMA block in the formation of a stronger gel-network through the chelation of Ca^2+^ cations to the carboxylate groups.

Applying a Z ratio of 1.00 to BCP 1:1 did not yield a gel-like material. To gain insight into the solution behavior of the block copolymer, DLS and TEM analyses were conducted (Fig. 5 and S2). DLS analysis of BCP 1:1 indicated an increase in hydrodynamic diameter from 3.7 nm (at Z = 0) to 24.5 nm (Z = 1.0) as unimeric block copolymer chains aggregated with increasing Ca ^2+^ chelation (Fig. 5A & Fig. S2a). TEM analysis (Fig. 5 D–F) confirmed aggregate formation (ca. 20 nm) for Z ratios between 0.6 and 1.0.

Attempts to make a gel at optimal conditions for BCP 1:1 (i.e. 7.5 % (w/v) and Z ratio of 1.57) utilizing BCP 1:2 were unsuccessful, as BCP 1:2 aggregated but remained in solution (Fig. 5 A–C and Fig. S3a–b). Varying the Z ratio between 0 and 2.0 resulted in the formation of aggregates with diameters between 17–29 nm, as evidenced by DLS and TEM (Fig. 5 A–C, Fig. S2B). Applying a Z ratio in excess of 2 and a BCP solid content of 15 % (w/v) still could not induce gelation. Instead, a high viscosity free-flowing solution was formed. It was concluded that the block ratio (with the lowest composition of the chelating P*t*BuSMA block and highest composition of the hydrophilic PNAM block) had rendered the 1:2 BCP too hydrophilic overall. Despite the increasing hydrophobicity of the P*t*BuSMA-Ca^2+^ component, the effective stabilization provided by the PNAM component due to its favorable interaction with water prevented the formation of the polymeric network required for gel formation (Fig. 4c). This suggests that it is essential to have an appropriate hydrophobic/hydrophilic balance along the BCP backbone if successful gelation is to be achieved.

**Fig. 6:**
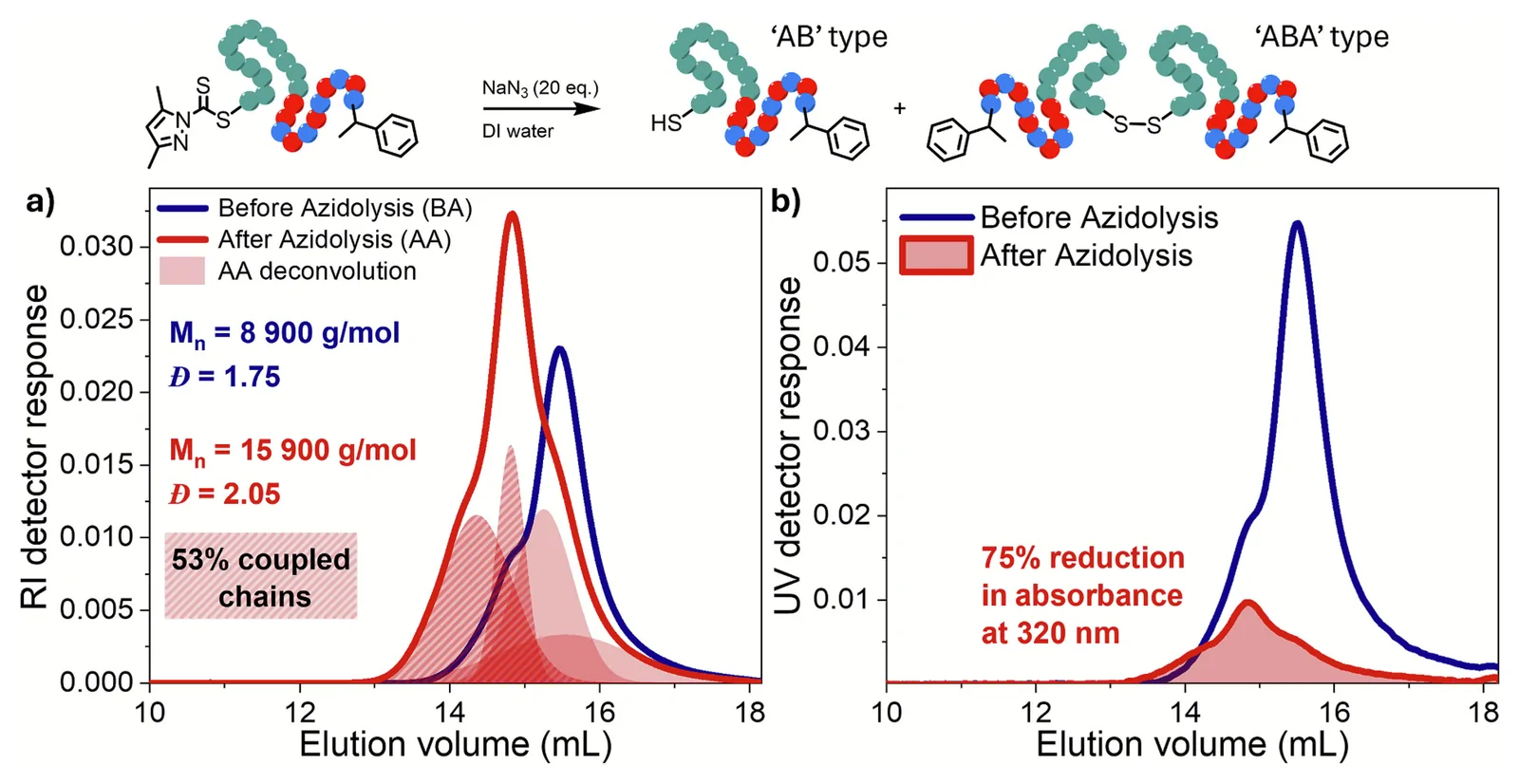
SEC analysis (DMF, 2 mM LiBr mobile phase) before and after azidolysis of BCP 1:1, including the deconvolution of RI eluograms (a) and UV eluograms (b) to estimate the proportion of coupled block copolymer chains and the extent of thiocarbonylthio removal, respectively.

Block copolymer architecture has been shown to influence the mechanism of gelation significantly. This has been demonstrated by Cui *et al*., using the thermo-gelation of AB-, ABA- and BAB-type block co-polymers constituting poly(D,L-lactide-*co*-glycolide)/PLGA (hydrophobic A block) and PEG (hydrophilic B block) [61]. For AB- and BAB-type block copolymers, thermo-gelation was facilitated by the formation of hydrophobic channels between semi-bald micelles. For ABA-type block copolymers, however, thermo-gelation proceeded *via* two possible pathways involving the formation of hydrophilic bridges or hydrophobic channels between semi-bald micelles. The present study aimed to assess briefly the effect of altering the architecture of P*t*BuSMA-*b*-PNAM on the mechanical properties of BCP gels. This is particularly relevant considering all di-block copolymer samples constitute a small proportion (22–36 %) of diverse copolymer architectures derived from chain transfer reactions (Fig. S1), discussed *vide supra*. It has been reported that sodium azide could be utilized to effectively convert thio- carbonylthio moieties into thiol groups, similar to end group removal strategies that employ amines [62,63]. The oxidation of thiol chain ends results in the coupling of polymer chains *via* S-S disulfide linkages, affording an ABA-type block copolymer with P*t*BuSMA acting as the hydrophobic A-block (upon complexation to Ca^2+^) and PNAM acting as the hydrophilic block (Fig. 6) [64]. BCP 1:1 underwent azidolysis under ambient conditions in water. Thereafter, BCP 1:1 and its azidolyzed derivative were protonated to facilitate dissolution in DMF (2 mM LiBr) for SEC analysis. UV detection at 320 nm indicated that azidolysis had been near quantitative (75 % reduction in peak area, Fig. 6b). Polymers with carboxylic acid functional repeat units are challenging to analyze as they generally interact with the column, resulting in tailing at high elution volumes (Fig. 6a). Additionally, the different mobile phase and calibration standards applied resulted in overall higher M_n_ (8 900 g/mol) and *Ð* (1.75) compared to that reported in Table 1. Nevertheless, comparison of the RI eluograms (Fig. 6a) show that the molecular weight of the BCP had approximately doubled after azidolysis, with deconvolution of the RI detector response indicating that 53 % of the distribution could correspond to BCP chains which have undergone disulfide coupling. Therefore, the sample after azidolysis constitutes a proportion of ABA-type DHBCs (i.e. P*t*BuSMA-*b*-PNAM-*b*-P*t*BuSMA); a copolymer similar in chemical composition to BCP 1:1, but with a different architecture.

**Fig. 7:**
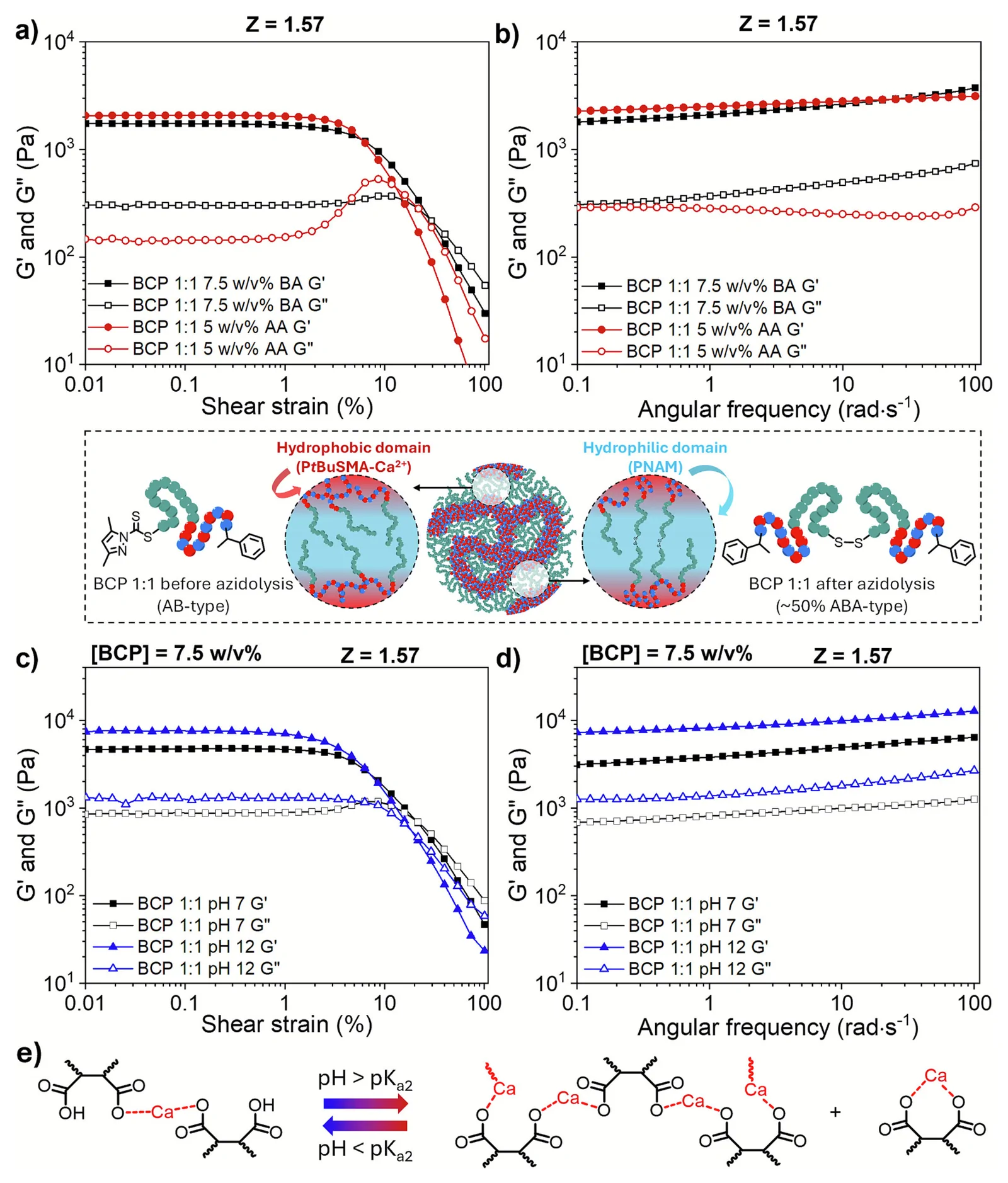
Amplitude **(a)** and frequency **(b)** sweeps of P*t*BuSMA-*b*-PNAM BCP 1:1 gels before and after azidolysis (BA and AA, respectively). Different BCP concentrations (5 and 7.5 % (w/v)) at a fixed Z ratio (1.57) were applied. Amplitude **(c)** and frequency **(d)** sweeps of BCP 1:1 gels at different pH values (7 and 12) and a fixed BCP concentration (7.5 % (w/v)) and Z ratio (1.57). Amplitude and frequency sweep experiments were performed at constant angular frequency (10 rad⋅s^-1^) and strain amplitude (1 %). The ionization state of maleic acid repeat units above and below pK_a2_ is represented in **(e)**.

Gelation experiments were subsequently conducted to form BCP gels, applying the previously optimized conditions (ambient conditions, 7.5 % w/v and Z = 1.57). Fig. 7 depicts the amplitude (Fig. 7a), as well as frequency (Fig. 7b) sweeps for these BCP gels. At 15 % (w/v), the azidolyzed BCP (with only a proportion of ABA-type BCPs) could not be fully dissolved, instead it formed a viscous solution prior to complete dissolution, precluding its use in gelation experiments. The azidolyzed BCP could be more amenable to forming chain entanglements, due to a combination of increased molecular weight and different block architecture, which significantly increased the viscosity of these solutions [65]. This practical challenge informed the decision to use the azidolyzed BCP under ambient conditions, as opposed to maximizing the proportion of ABA-type BCPs *via* oxidation of thiol chain ends. The solid content of the thiol-terminated BCP was consequently reduced to 10 % (w/v) (i.e. 5 % w/v final solid content after addition of the Ca^2+^ solution), to try and achieve complete dissolution. Indeed, the BCP dissolved and the Ca^2+^ solution was successfully added (Fig. S4a).

The introduction of Ca^2+^ gave rise to BCP gels (5 % w/v and Z = 1.57) whose mechanics proved superior to BCP 1:1 before azidolysis (7.5 % w/v and Z = 1.57, Fig. 7). This was an interesting observation, given that the solid content of the azidolyzed copolymers was significantly less (by approximately 33 %) than its pre-azidolysis counterpart, which highlights the effect of BCP architecture on the mechanical properties of the gel. Attempts to prepare a corresponding BCP 1:1 gel (5 % w/v and Z = 1.57) were unsuccessful, yielding a slightly viscous solution (Fig. S4b) as these conditions represent the onset of gelation, and the resulting material is characterized by slow gelation kinetics. This is consistent with the results presented in Fig. 3a, where a Z ratio of 1.88 was required at 5 % (w/v) to form a gel that can be reliably measured. BCP 1:1 (after azidolysis) has a proportion of ABA-type copolymers which could contribute hydrophilic PNAM bridges which connect hydrophobic P*t*BuSMA-Ca^2+^ domains more effectively than AB-type co-polymers, thereby affording a gel at significantly lower solid content (Fig. 7).

Up to this point, the BCP gelation experiments were conducted in a Tris HCl buffer solution (pH 7.6, 50 mM). P*t*BuSMA maleic acid repeat units (with pK_a1_ = 5.6 and pK_a2_ = 8.0) are partially ionized when the pH is between pK_a1_ and pK_a2_ (Fig. 7c) and therefore have approximately one carboxylate moiety per repeat unit (α = 1) for intra- or intermolecular chelation with calcium. At pH > pK_a2_ the maleic acid repeat units are fully ionized (α = 2), providing additional sites for chelation with Ca^2+^. To investigate the effect of pH on the gel properties (Fig. 7 c–d), gelation experiments were conducted at pH 7 (< pK_a2_) and 12 (> pK_a2_) in DI water. Protonation of the carboxylate pendant groups increases the overall hydrophobicity of the P*t*BuSMA block and additionally, facilitates hydrogen bond formation with the PNAM block [52]. Therefore, conducting these experiments at pH values below 7 proved challenging, owing to the BCP’s propensity to crash out of solution in acidic conditions [52]. BCP 1:1 gels prepared at both pH values exhibited good mechanical strength, but pH 12 favored the formation of stiffer gels. The provision of additional chelation sites likely increases the probability of forming more carboxylate-Ca^2+^ complexes between BCP chains (for a given Z ratio) which translates to increased crosslink density, and thus stiffer BCP gels.

**Fig. 8:**
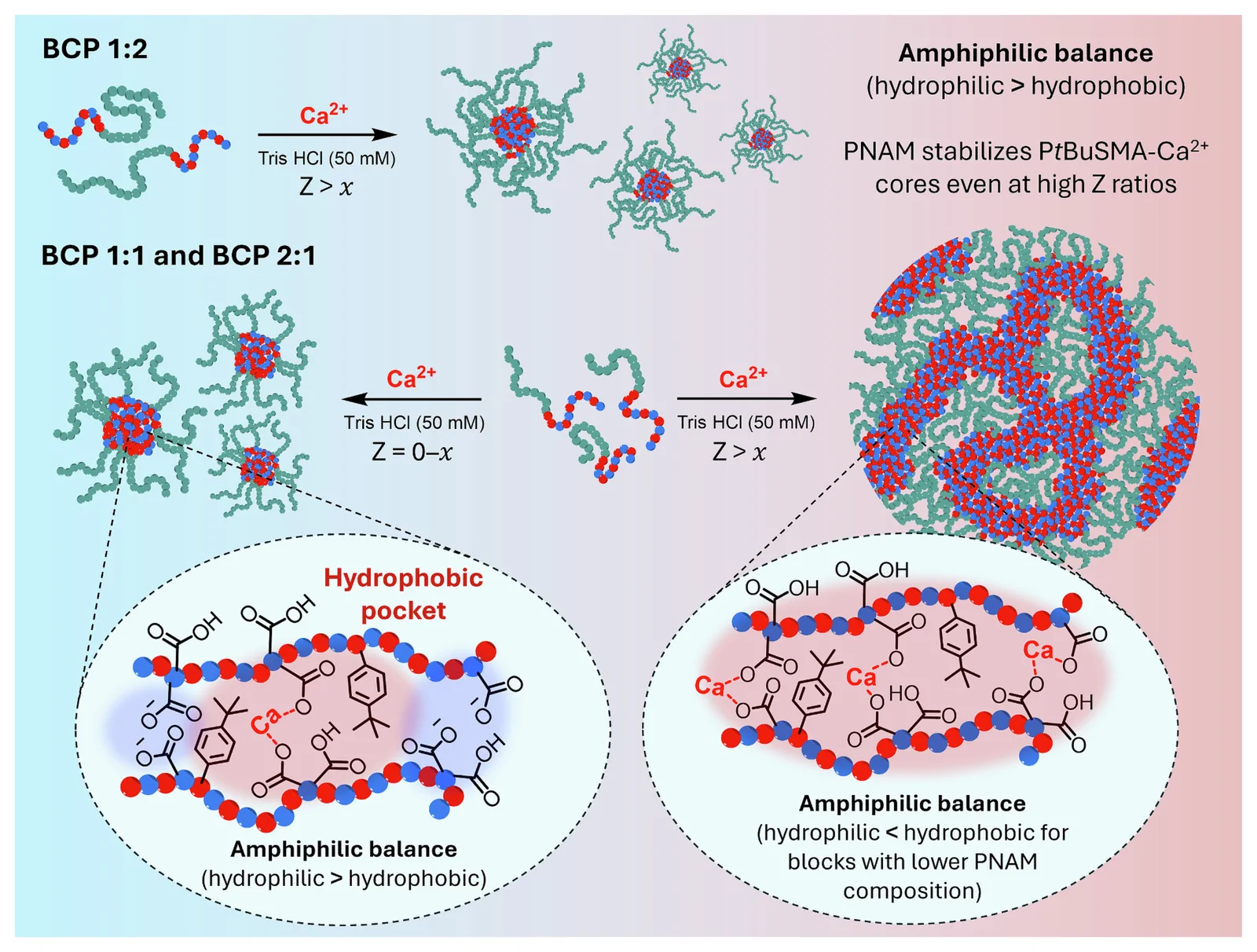
Graphic representation of factors such as block ratio, Z ratio and amphiphilic balance which influence gel formation for P*t*BuSMA-*b*-PNAM block copolymers when treated with Ca^2+^ under appropriate conditions.

Overall, the data suggests that the different ions (other than Ca^2+^) present in the Tris HCl buffer solution do not significantly contribute towards gelation (Fig. S5). Moreover, previous reports concerning the formation of P*t*BuSMA-*b*-PNAM/Ca^2+^ gels would suggest that the presence of the bulky hydrophobic *tert*-butyl group and the large cationic radius of Ca^2+^ play an important role [52]. Klumperman and co-workers showed that omission of the *tert*-butyl group (through utilization of PSMA-*b*-PNAM-Ca^2+^) or using a divalent cation with smaller ionic radius (i.e. P*t*BuSMA-*b*-PNAM-Mg^2+^) did not facilitate the formation of a gel but instead promoted micelle formation [52]. This underscores the crucial role played by the bulky hydrophobic *tert*-butyl group on the P*t*BuSMA block in the gelation process. We postulate that through hydrophobic effects, physical crosslinks are spontaneously formed in the hydrophobic domains in a quest to minimize their exposure to the aqueous environment (Fig. 8) [66]. In addition, smaller Mg^2+^ cations have a high water-binding energy relative to larger Ca^2+^ cation, which would explain the latter’s propensity to rather chelate to the negatively charged carboxylate groups on the P*t*BuSMA block [67,68]. In light of this, it appears as if chelation and the subsequent formation of hydrophobic domains are the primary contributors towards the gelation mechanism, since the BCP gels displayed the highest stiffness at pH 12.

Based on the data presented herein, it is evident that the amphiphilic balance of the P*t*BuSMA-Ca^2+^ and PNAM components (Fig. 8**)**, as well as BCP architecture, also influence the gelation process significantly. Hydrophobic interactions were fundamental in facilitating the gelation process, given that gelation was not achieved in the absence of the hydrophobic *tert*-butyl group (i.e. for PSMA-*b*-PNAM/Ca^2+^), [52] or where *tert*-butyl groups are present but proportion of the hydrophilic PNAM component is too high (i.e. the 1:2 BCP). Despite the formation of very hydrophobic P*t*BuSMA-Ca^2+^ domains at high Z ratios (Z > *x*, Fig. 8), the amphiphilic balance resulting from the BCP 1:2 block ratio favors micelle formation. For BCPs with a smaller PNAM component (i.e. BCP 1:1) this amphiphilic balance is still crucial for successful gel formation, as insufficient calcium (Z = 0–*x*, Fig. 8) promotes micelle formation, while gel formation could only be achieved through increased hydrophobicity of the P*t*BuSMA-Ca^2+^ component (Z > *x*, Fig. 8). For BCP 2:1 (with the smallest PNAM component), gelation could be achieved at much lower Z ratios, owing to the dominating contribution of the chelating P*t*BuSMA block in tipping the amphiphilic balance in favor of gelation and not micelle formation.

**Fig. 9:**
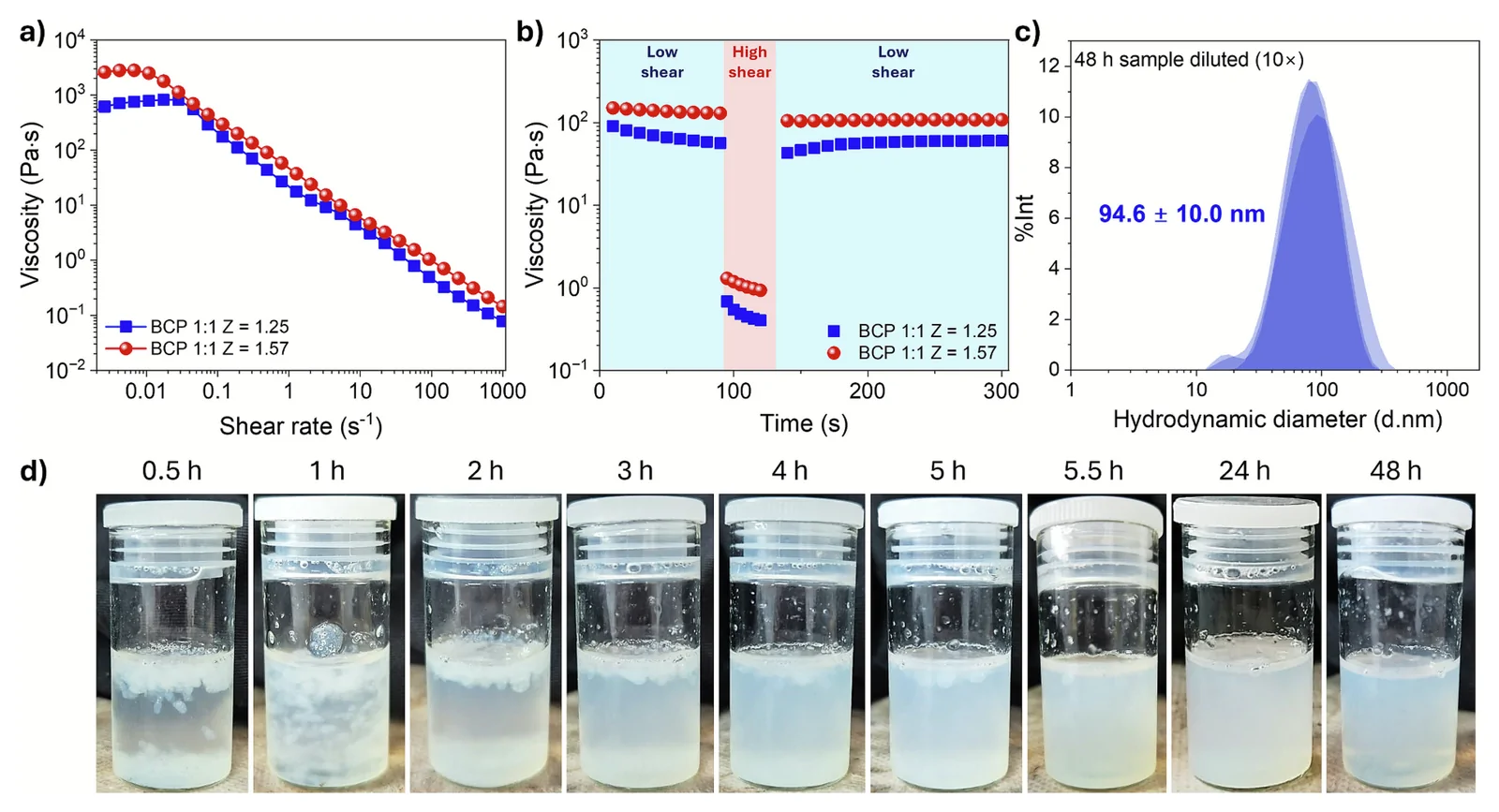
Flow curves **(a)** and thixotropy data **(b)** for the BCP 1:1 gels at 7.5 % (w/v) and Z ratios of 1.25 and 1.57. For the thixotropy tests, applied shear rates were 0.25 s^−1^ for the first and third, low shear intervals, and 100 s^−1^ for the second, high shear interval. A representative BCP 1:1 gel (7.5 % w/v, Z = 1.57, 1 mL) was injected into Tris HCl buffer (5 mL) at room temperature, allowed to rest relatively undisturbed for 48 h and subsequently an aliquot of the solution was analyzed *via* DLS **(c)**. The dissolution of the gel was tracked visually in **(d)**.

To broaden the scope of applicability of these BCP-based gels, particularly in drug delivery systems, where injectability is beneficial, we assessed their behavior under high shear conditions (Fig. 9a) [69]. Under low shear, BCP 1:1 at Z = 1.57 has a higher shear viscosity than when Z = 1.25, consistent with the results presented in Fig. 3 c–d, where the elastic modulus was higher at this Z ratio. The shear viscosity of both BCP gels displayed a strong dependence on shear rate, notably when a higher shear force was applied, decreasing markedly with an increase in shear rate in the assessed shear rate range (Fig. 9a). This behavior is typical of non-Newtonian fluids, whose viscosity depends on the applied stress [70]. The observed shear-thinning character suggests that these BCP gels could potentially be employed as injectable materials, if they have self-healing properties.

In light of this, a thixotropy step test was conducted to assess the self-healing capabilities of these BCP gels (Fig. 9b). The first and the third intervals are low shear intervals (0.25 s^−1^ shear rate), mimicking “rest conditions” and the second interval is a high shear interval that simulates the high shear forces associated with injection (100 s^−1^). Baseline properties in the first interval also displayed higher shear viscosity for the BCP gels where Z = 1.57 compared to where Z = 1.25, the shear viscosity of both gels significantly dropped in the second, high shear interval, as the applied shear force was sufficient to cause disruption of the gel network, consistent with the pseudo-plastic behavior observed in Fig. 9a. Interestingly, the shear viscosity of the BCP gels was almost instantly restored in the third, low shear interval upon cessation of applied high shear. The observed structural regeneration could be ascribed to the reorganization of the hydrophobic domains and formation of new transient linkages due to the dynamic nature of these BCP gels [71]. Although instantaneous elastic recovery is evident in the third interval, it is expected that the recovery is not complete, as a small portion of strain remains within these viscoelastic materials, owing to partial permanent deformation caused by time-dependent viscous flow.

The injectability of a BCP 1:1 gel (7.5 % w/v, Z = 1.57) was then assessed. A gel was prepared by drawing up 0.5 mL of polymer solution (15 % w/v in Tris HCl buffer) into a 3 mL syringe, followed by 0.5 mL of Ca^2+^ solution (160 mM in Tris HCl buffer), causing rapid gelation to occur inside the syringe. The next day, the gel was ejected from the syringe (fitted with a 40 mm, 20G needle) into 5 mL of Tris HCl buffer (see supplementary movie S1) and stored at 22 °C for 48 h. The gel initially appeared to maintain its structure upon injection but with time dissolution of the gel was observed (Fig. 9d). After 48 h, an aliquot of the solution (1.25 % w/v polymer, Z = 1.57) was diluted and analyzed *via* DLS (Fig. 9c), indicating that P*t*BuSMA-*b*-PNAM/Ca^2+^ aggregates (ca. 95 nm) had formed. This was consistent with previous findings, which employed similar polymer and calcium concentration [52]. It was hypothesized that the dissolution of the gel was facilitated primarily by the diffusion of calcium from the gel structure, similarly to the materials reported by Nakagawa *et al*. [45]. The injection experiment described in Fig. 9d was repeated, but the gels were injected into Tris HCl buffer with the same concentration of calcium as the gel (80 mM, see supplementary movie S2). Consequently, the gel dissolved at a much slower rate and large sedimenting gel particles could still be observed 24 h post-injection (Fig. S6). This would suggest that the ionic strength and calcium composition of the injection medium significantly impact the gel’s dissolution kinetics.

## 3 Conclusions

Owing to the biomedical potential of P*t*BuSMA-*b*-PNAM/Ca^2+^ materials, various parameters which influence the gelation of these complexes were systematically investigated. Increasing the concentration of P*t*BuSMA-*b*-PNAM improved gel mechanical properties, as the availability of more BCP chains facilitated the formation of a denser gel network. Higher crosslinking density could be achieved by increasing the concentration of Ca^2+^, resulting in the formation of gels with higher stiffness. Ultimately, a BCP concentration of 7.5 % (w/v) and a Z ratio of 1.57 were found to produce BCP 1:1 gels with optimal properties. These conditions were applied to P*t*BuSMA-*b*-PNAM block copolymers with different block ratios (i.e. 1:1, 2:1 and 1:2). BCP 2:1 gels (with the highest composition of P*t*BuSMA) exhibited superior mechanical properties to BCP 1:1 gels and BCP 1:2 (with the highest composition of PNAM) did not form gels. This emphasized the crucial role played by the chelating P*t*BuSMA block in the formation of a stronger gel-network and the importance of an appropriate overall amphiphilic balance in P*t*BuSMA-*b*-PNAM/Ca^2+^ complexes. Azidolysis of BCP 1:1 was conducted to afford a BCP sample with higher molecular weight and altered block copolymer architecture, but similar chemical composition. The azidolyzed sample formed a gel with superior mechanical properties relative to BCP 1:1 (before azidolysis), allowing for the preparation of gels at much lower polymer concentration. The effect of pH was briefly assessed, whereby the provision of additional chelation sites, due to the complete ionization of MA repeat units at elevated pH, resulted in gels with higher crosslink density and improved mechanical properties. Lastly, the behavior of BCP 1:1 gels (at 7.5 % w/v and Z = 1.25–1.57) under high shear conditions and during a thixotropy step test was assessed. The observed shear-thinning character and the indication of self-healing properties suggested that these P*t*BuSMA-*b*-PNAM/Ca^2+^ gels could be promising candidates for injectable materials.

## 4 Experimental

### 4.1 Materials

4-*tert*-Butyl-styrene (*t*BuSTY, Merck, 93 %, stabilized with *tert*-butylcatechol) was eluted from an aluminum oxide (Merck, activated basic, Brockmann I) column, prior to use. Maleic anhydride (MAnh, Merck, 99 %) and 1,3,5-trioxane (Merck, 99 %) were recrystallized from distilled chloroform, filtered and dried under vacuum or alternatively sublimed as needed. 4-Acryloylmorpholine (NAM, stabilized with 1000 ppm monomethyl ether hydroquinone, Merck, 97 %) was vacuum distilled before use. Azobisisobutyronitrile (AIBN, Merck, 98 %) was recrystallized from anhydrous methanol, filtered and dried under vacuum⋅THF was pre-dried over KOH pellets, filtered and distilled over sodium/benzophenone. Tris HCl buffer (1 M, UltraPure™, Invitrogen) was diluted to 50 mM (*p*H 7.6) using DI water. MgCl_2_⋅6H_2_O (Merck, ≥ 99 %), CaCl_2_ (Merck, ≥ 99 %), KOH (Merck, 90 %, flakes), CS_2_ (Kimix, 99 %), 3,5-dimethylpyrazole (Merck, 99 %), 1-bromoethyl benzene (Merck, 97 %), 1,4-dioxane (Merck, anhydrous, 99.8 %), acetone (Merck, ≥ 99.5 %), NaOH (Merck, pellets for analysis) and 3500 MWCO SnakeSkin™ dialysis tubing (Thermo Fisher Scientific) were used as received.

## 5 Methods

### 5.1 Synthesis of PtBuSMAnh macro-CTAs

The CTA was synthesized according to previously reported procedures, with similar yield and purity obtained [52,57]. Considering the synthesis of macro-CTA**1** as a representative protocol, *t*BuSTY (24.6 g, 153 mmol), MAnh (10.0 g, 102 mmol), 1-phenylethyl 3,5-dimethyl-1*H*-pyrazole-1-carbodithioate (1.41 g, 5.10 mmol), AIBN (84.5 mg, 0.515 mmol), 1,3,5-trioxane (194 mg, 2.16 mmol) and 1,4-dioxane (120 mL) were added to a round bottom flask fitted with a magnetic stirrer bar, rubber septa and bubbler. The reaction mixture was sparged with dry argon for 1 h and subsequently immersed in an oil bath preheated at 60 °C for 22 h. The reaction mixture was then cooled, diluted with acetone (ca. 20–50 mL) to reduce the viscosity of the solution and the copolymer precipitated in pentane (ca. 1–1.5 L). The copolymer was filtered and dried under vacuum at ambient temperature for 24 h.

### 5.2 Synthesis of PtBuSMAnh-b-PNAM BCPs

Considering the synthesis of BCP 1:1 as a representative protocol, P*t*BuSMAnh (macro-CTA**1**, 21.7 g, 3.57 mmol), NAM (30.7 g, 218 mmol), AIBN (59.3 mg, 0.361 mmol), 1,3,5-trioxane (320 mg, 3.55 mmol) and 1,4-dioxane (175 mL) were added to a round bottom flask fitted with a magnetic stirrer bar, rubber septa and bubbler. The reaction mixture was sparged with dry argon for 1 h and subsequently immersed in an oil bath preheated at 60 °C for 20 h. The reaction mixture was then cooled, diluted with DCM (ca. 20–50 mL) to reduce the viscosity of the solution and the copolymer precipitated in pentane (ca. 2 L). The copolymer was filtered and dried under vacuum at ambient temperature for 24 h.

### 5.3 Hydrolysis of PtBuSMAnh-b-PNAM BCPs

The hydrolysis of BCP 1:1 is briefly described, whereby a similar procedure was applied for BCP 1:2 and BCP 2:1. For the hydrolysis of BCP 1:1, the block copolymer (48 g, 43 % w/w MAnh) was dissolved in THF (160 mL) and added to a solution of NaOH (3.20 g, 1.0 eq with respect to MAnh units) in DI water (320 mL) preheated to 60 °C for 0.5 h. The solution was cooled and transferred to dialysis tubing (MWCO 3500) and dialyzed against DI water for 3 days (with fresh DI water exchanges occurring 2–3 × daily). The pH of the hydrolyzed block copolymer was measured (8.41) and BCP 1:1 was subsequently isolated *via* lyophilization. The pH of BCP 1:2 and BCP 2:1 prior to lyophilization was 9.27 and 9.00 respectively. Successful hydrolysis was confirmed *via* ATR-FTIR spectroscopy.

### 5.4 Azidolysis of BCP 1:1

BCP 1:1 (P*t*BuSMA-*b*-PNAM, 3.0 g, 0.2 mmol) in DI water (10 mL) was treated with NaN_3_ (0.3 g, 4.1 mmol) and stirred under ambient conditions (i.e. in the presence of atmospheric oxygen). The solution changed from pale yellow to colorless and exhibited a significant increase in viscosity after 10 h. The solution was diluted with DI water (ca. 20 mL), transferred to dialysis tubing (3500 MWCO) and dialyzed with DI water under ambient conditions (replaced 2–3 × daily for 2 days). The pH of the polymer solution was then adjusted to approximately 8 and lyophilized. The isolated block copolymer was characterized *via* SEC (with RI and UV detection) to confirm that azidolysis had been successful.

### 5.5 Gelation experiments

An exemplary protocol (outlined in Scheme 1) for the gelation of BCP 1:1 at 7.5 % (w/v) and Z = 1.57 (i.e. 80 mM Ca^2+^) entails dissolving P*t*BuSMA-*b*-PNAM (${\text{M}}_{\text{n}}^{\text{theo}\;}=14700\text{g}\cdot {\text{mol}}^{-1}\;$, 0.225 g, 15.3 μmol) in Tris HCl buffer (1500 μL, 50 mM) in one scintillation vial for an initial BCP concentration of 15 % (w/v). In another vial, a dilute Ca^2+^ solution was prepared by adding Tris HCl buffer (900 μL, 50 mM) to a Ca^2+^ stock solution (600 μL, 0.4 M). For final BCP and Ca^2+^ concentrations of 7.5 % (w/v) and 80 mM, respectively, the dilute Ca^2+^ solution was added to the BCP solution *via* a glass pipette while agitating the vial to promote rapid mixing of the two solutions. A gel-like material formed within seconds and the contents of the vial ceased to flow.

### 5.6 Characterization

#### 5.6.1 NMR spectroscopy

NMR spectroscopic analyses were carried out using a 400 MHz/600 MHz Bruker Ascend Spectrometer, specified per sample. Samples were dissolved in either (CD_3_)_2_SO (Merck, Magni-Solv™, 99.9 %) or D_2_O (Merck, Magni-Solv™, 99.9 %) prior to analysis, specified per sample.

#### 5.6.2 ATR-FTIR spectroscopy

Attenuated total reflectance infrared (ATR-FTIR) spectroscopy was performed using a Thermo Scientific Nicolet iS10 Smart iTR, using 128 scans over the wavelength range of 600–4000 cm^−1^, with a background spectrum (64 scans) obtained prior to each sample analyzed.

#### 5.6.3 SEC

Size exclusion chromatography was conducted using two different systems, specified per sample. The first protocol employed THF (5 % v/v AcOH with 0.125 % BHT, Merck, for HPLC, ≥99.9 %) with samples dissolved at 2 mg/mL and filtered using 0.45 µm RC filters (Sartorius) prior to analysis. The analysis was performed on an Agilent 1260 HPLC instrument fitted with a quaternary pump, a column compartment thermostated at 30 °C, a differential refractometer set at 30 °C and a diode array UV detector set at 254 and 320 nm. The columns utilized were two Agilent Technologies PLgel 5 Mixed-C columns (300 × 7.5 mm i.d.) and a PLgel 5 Guard column (50 × 7.5 mm i.d.). The flow rate during analysis was 1.0 mL/min and the injection volume per sample was 100 µL. The system was calibrated using low *Ð* PS calibration standards with a molar mass range of 580–2.0 × 10^6^ g/mol. The second protocol employed LiBr stabilized DMF (2 mM, Merck, Chromosolv® Plus, for HPLC, ≥ 99.9 %), with samples acidified, lyophilized and subsequently dissolved at 2 mg/mL prior to analysis. The samples were filtered using 0.45 µm PTFE filters (Sartorius) prior to analysis with an Agilent 1260 HPLC instrument fitted with a quaternary pump, thermostated column compartment set at 60 °C, an autosampler, a differential refractometer set at 50 °C and a diode array UV detector set at 320 nm. Columns utilized were Agilent PLgel Mixed-C (5 µm) guard column (50 × 7.5 mm i.d.) and two analytical columns (300 × 7.5 mm i.d.). The flow rate during analysis was 1.0 mL/min and the injection volume per sample was 100 µL. RAFT-synthesized PSMAnh calibration standards (600–90 000 g/mol) were utilized.

#### 5.6.4 Dynamic light scattering (DLS)

DLS analyses, for assessment of the BCP aggregates, were conducted using a ZetaSizer 1000 HSa (Malvern Instruments, Malvern), fitted with a 4 mW He-Ne laser, operating at a wavelength of 633 nm and a scattering angle of 90°. Analyses and data processing were conducted using ZetaSizer Software 8.

#### 5.6.5 Transmission electron microscopy (TEM)

TEM analyses for BCP aggregates were undertaken using a JEOL 1200 EX microscope, equipped with a Gatan Orius CCD camera, with an accelerating voltage of 120 kV utilized. Prior to imaging, a 5 mg/mL DHBC solution was prepared (×10 dilution) and 1 μL was spotted onto a copper grid. The sample was stained with a 2 % uranyl acetate solution and subsequently dried at room temperature.

#### 5.6.6 Rheometry

To determine the mechanical properties of BCP gels, rheological measurements were performed using an Anton Paar GmbH (MCR302) rheometer. The instrument was fitted with a parallel plate geometry (diameter, 25 mm) and a measuring gap of 1 mm was used. Samples were placed on the bottom plate at 25 °C, the geometry was lowered to the measuring position and the hood was subsequently lowered to enclose the sample. Amplitude sweeps were performed at a constant angular frequency of 10 rad•s^−1^ and the amplitude of oscillation was varied between 0.01 and 100 %. Frequency sweeps were subsequently carried out in the 0.1–100 rad•s^−1^ angular frequency range, at fixed strain amplitudes (as determined by the limit of LVE region). Rheo- Compass software (version 1.2) was used for system control, data capturing and data processing.

## Supplementary Material

SI Supplementary data to this article can be found online at https://doi.org/10.1016/j.eurpolymj.2026.114504.

## Figures and Tables

**Table 1:** Monomer conversion and molecular weight data for macro-CTAs and P*t*BuSMAnh-*b*-PNAM block copolymers.

Entry	Sample	Ratio ^a^	[CTA + M] (w/v%) ^b^	α^MAnh^ (%) ^c^	α*^t^*^BuSTY^ (%) ^c^	α^NAM^ (%) ^c^	${\text{M}}_{\text{n}}^{\text{theo}\;}$	${\text{M}}_{\text{n}}^{\text{SEC}}$	*Đ* ^e^
**1**	macro-CTA**1**	30:20:1:0.1	30	100	81	–	6 100	7 000	1.24
**2**	macro-CTA**2**	55:45:1:0.2	25	100	94	–	13 000	15 600	1.32
**3**	BCP 1:1^f^	61:1:0.1	30	–	–	100	14 700	14 400	1.21
**4**	BCP 1:2	122:1:0.2	30	–	–	7	7 300	–	–
**5**	BCP 1:2^f^	122:1:0.4	40	–	–	100	23 300	20 300	1.21
**6**	BCP 2:1^f^	61:1:0.5	50	–	–	100	21 600	23 500	1.52

**(a)** Reagent ratio, [*t*BuSTY]:[MAnh]:[CTA]:[AIBN] for entries 1–2 or [NAM]:[macro-CTA]:[AIBN] for entries 3–5, **(b)** concentration of CTA and monomer in the polymerization reaction mixture (solvent, reaction temperature and time are 1,4-dioxane, 22 h and 60 °C, respectively for all reactions), **(c)** monomer conversion determined *via* ^1^H NMR spectroscopy using 1,3,5-trioxane as internal standard, **(d)** calculated using monomer conversion, **(e)** determined *via* SEC, using THF (5 % AcOH) as mobile phase and PS calibration standards, **(f)** block copolymers before alkaline hydrolysis of MAnh repeat units.

## Data Availability

Data will be made available on request.
