# Supplementary material for: Mechanistic insights into the calcium-mediated gelation of poly(4-tert-butylstyrene-alt-maleic acid)-block-poly(N-acryloylmorpholine) double hydrophilic block copolymers
Source: Eur Polym J. Author manuscript; Available in PMC 2026 Sep 15. (PMC7619492; doi:10.1016/j.eurpolymj.2026.114504)
Supplement: Supplementary file 1 [file EMS213188-supplement-SI.pdf]

Supplementary Information

Mechanistic insights into the calcium-mediated gelation of poly(4-*tert*-butylstyrene-*alt*-maleic acid)-*block*-poly(*N*-acryloylmorpholine) double hydrophilic block copolymers

Lauren E. Ball,*‡[a,b] Bennie Motloung,*‡[b] Michael-Phillip Smith,[b] Rueben Pfukwa[b] and Bert Klumperman*[b]

‡Both authors contributed equally to this manuscript

[a] Leibniz-Institut für Polymerforschung Dresden e.V., Hohe Straße 6, D-01069 Dresden, Germany

[b] Department of Chemistry and Polymer Science, Stellenbosch University, Private Bag X1, Stellenbosch 7599, South Africa

E-mail: ball@ipfdd.de, motloung@sun.ac.za and bklump@sun.ac.za

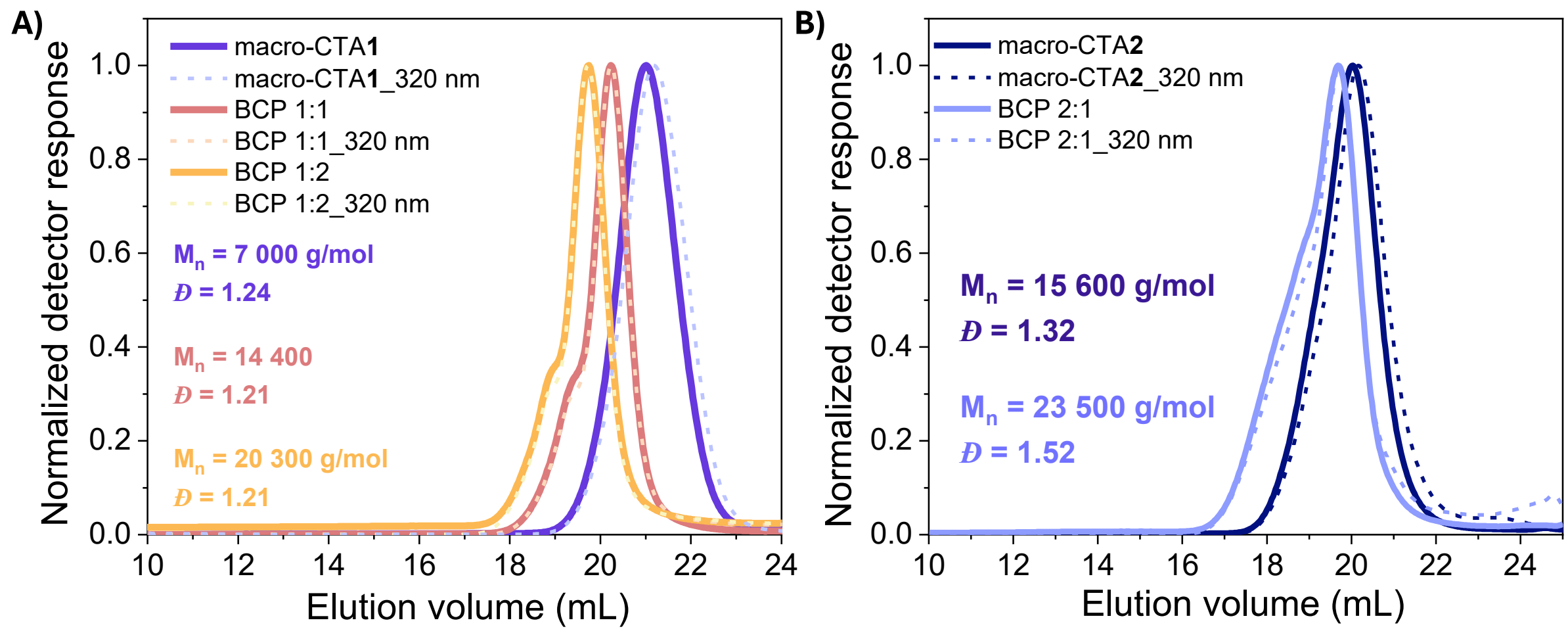

Figure S1. SEC analyses of macro-CTA**1**/**2** and corresponding block copolymers, using THF (5% (v/v) AcOH) as the mobile phase and low *Đ* PS calibration standards. Normalized data for both RI and UV (320 nm) detectors are shown. Deconvolution of the RI eluograms for all three block copolymers indicated that 24% (BCP 1:1), 22% (BCP 1:2) and 36% (BCP 2:1) of the distribution corresponded to high molecular weight copolymers, likely derived from chain transfer reactions.

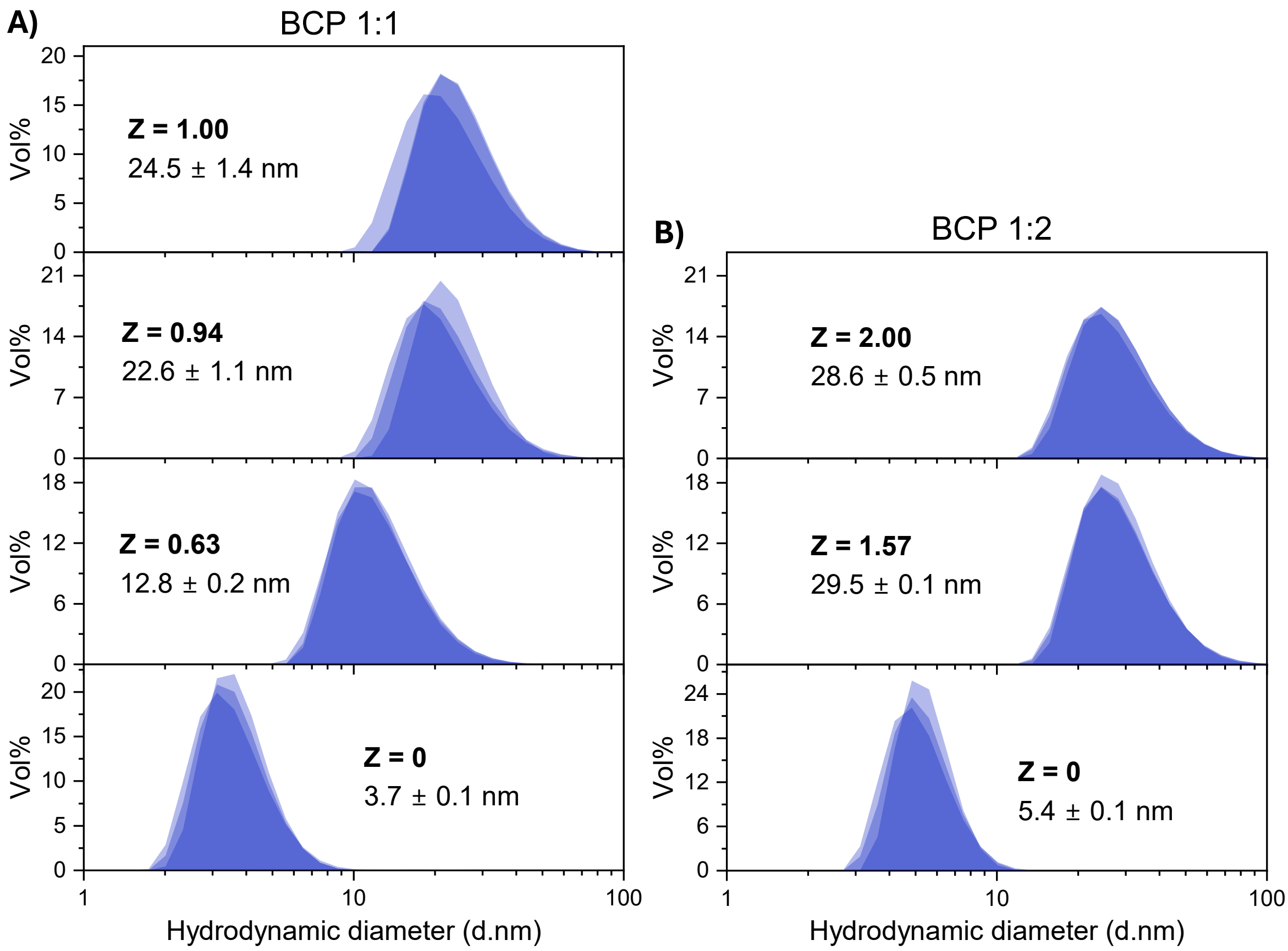

Figure S2. DLS analyses for BCP 1:1 (A) and BCP 1:2 (B) from Figure 5 of the main text. Samples were prepared at 7.5% (w/v) with varying Z ratio and subsequently diluted to 0.5% (w/v) prior to analysis (in triplicate).

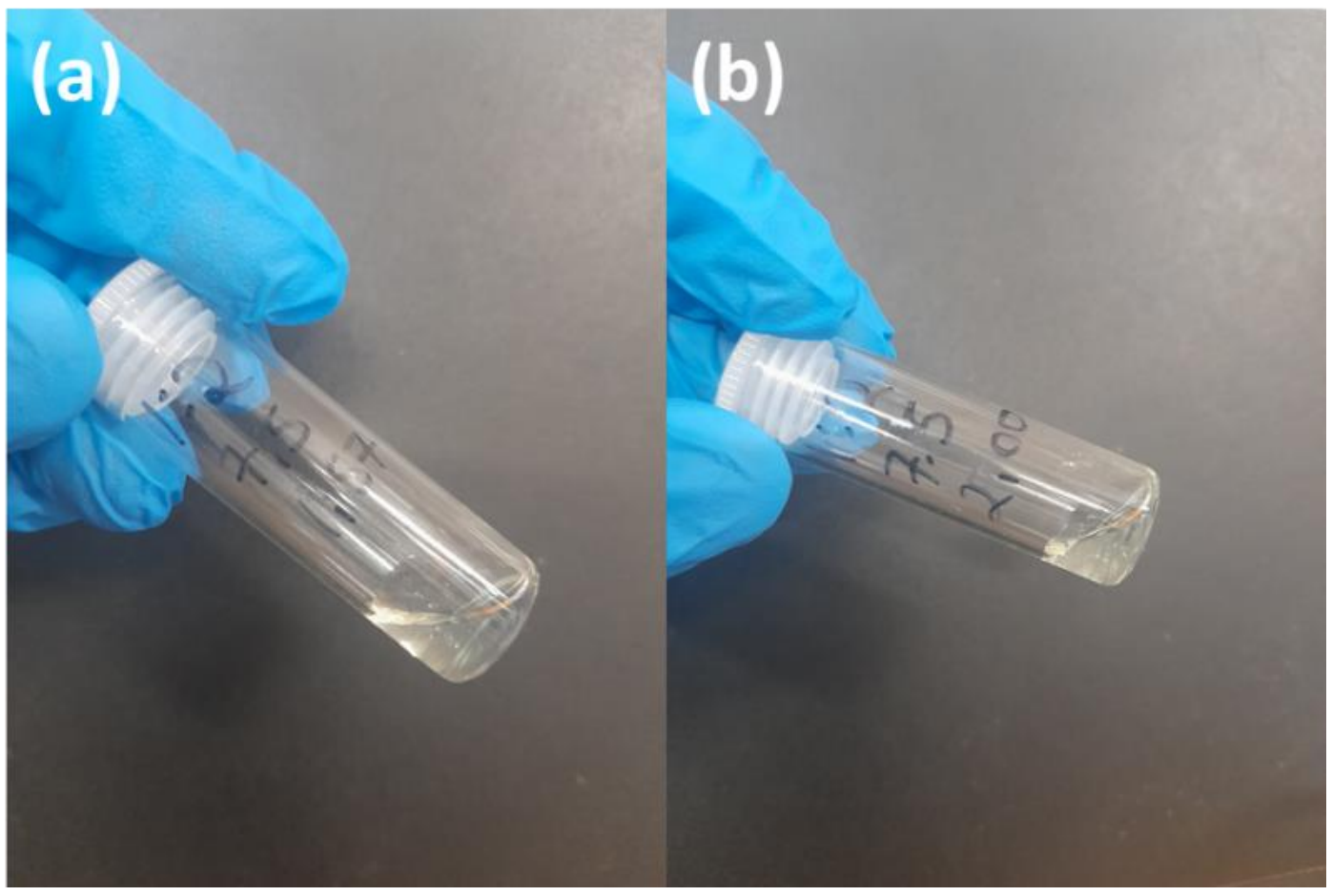

Figure S3. P*t*BuSMA-*b*-PNAM/Ca^{2+} aqueous solutions prepared using BCP 1:2 at 7.5% (w/v) and Z ratios of (a) 1.57 and (b) 2.00. Both BCP samples did not form a gel, however, the apparent solution viscosity was higher at Z = 2 compared to where Z = 1.57.

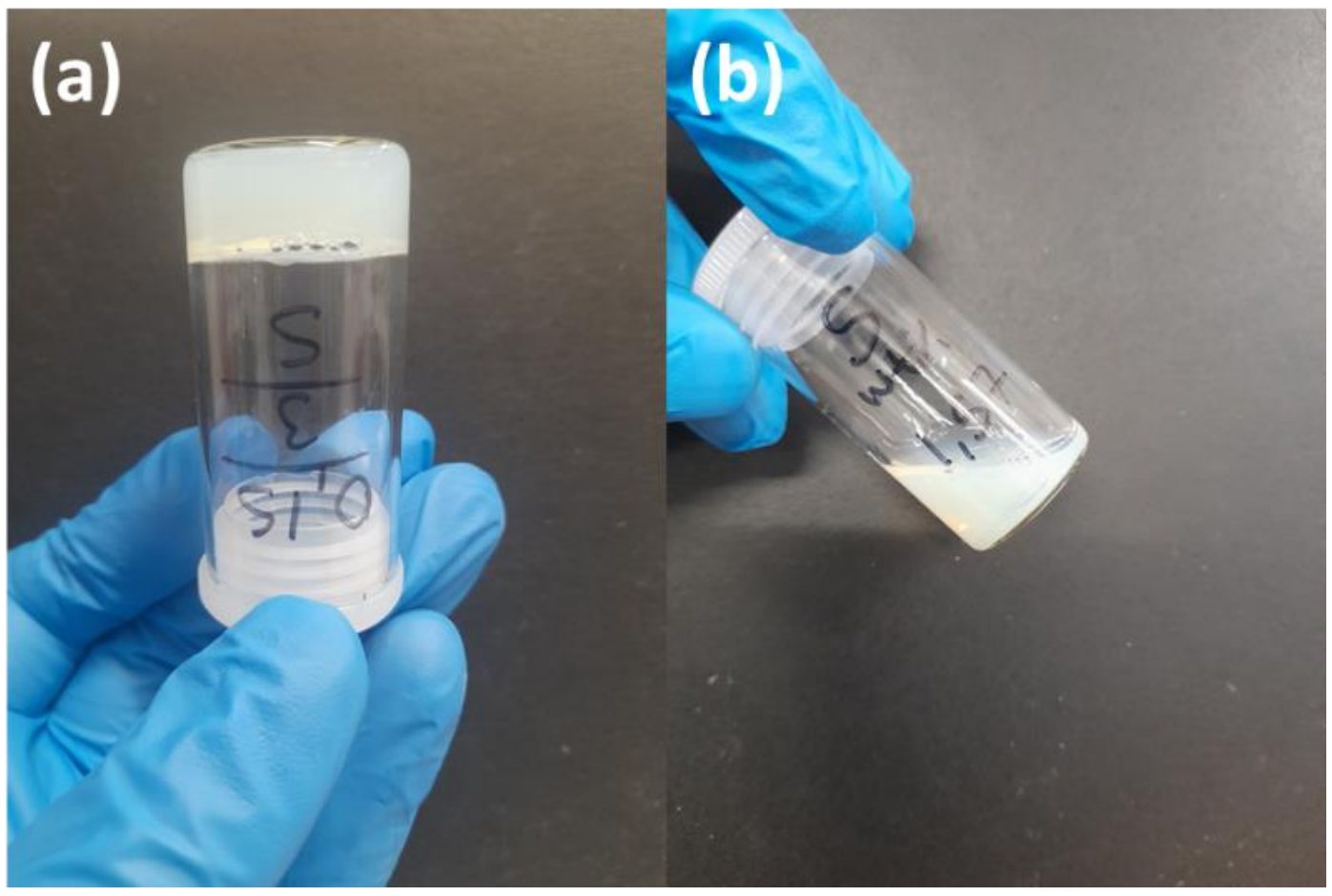

Figure S4. The effect of altering P*t*BuSMA-*b*-PNAM architecture on the gelation behavior of BCP 1:1 at 5% (w/v). Both BCP gels are prepared at 5% (w/v) and Z = 1.57, however, the azidolyzed BCP forms a gel (a), while the same results could not be achieved using the original BCP 1:1 copolymer (b) (i.e. without azidolysis).

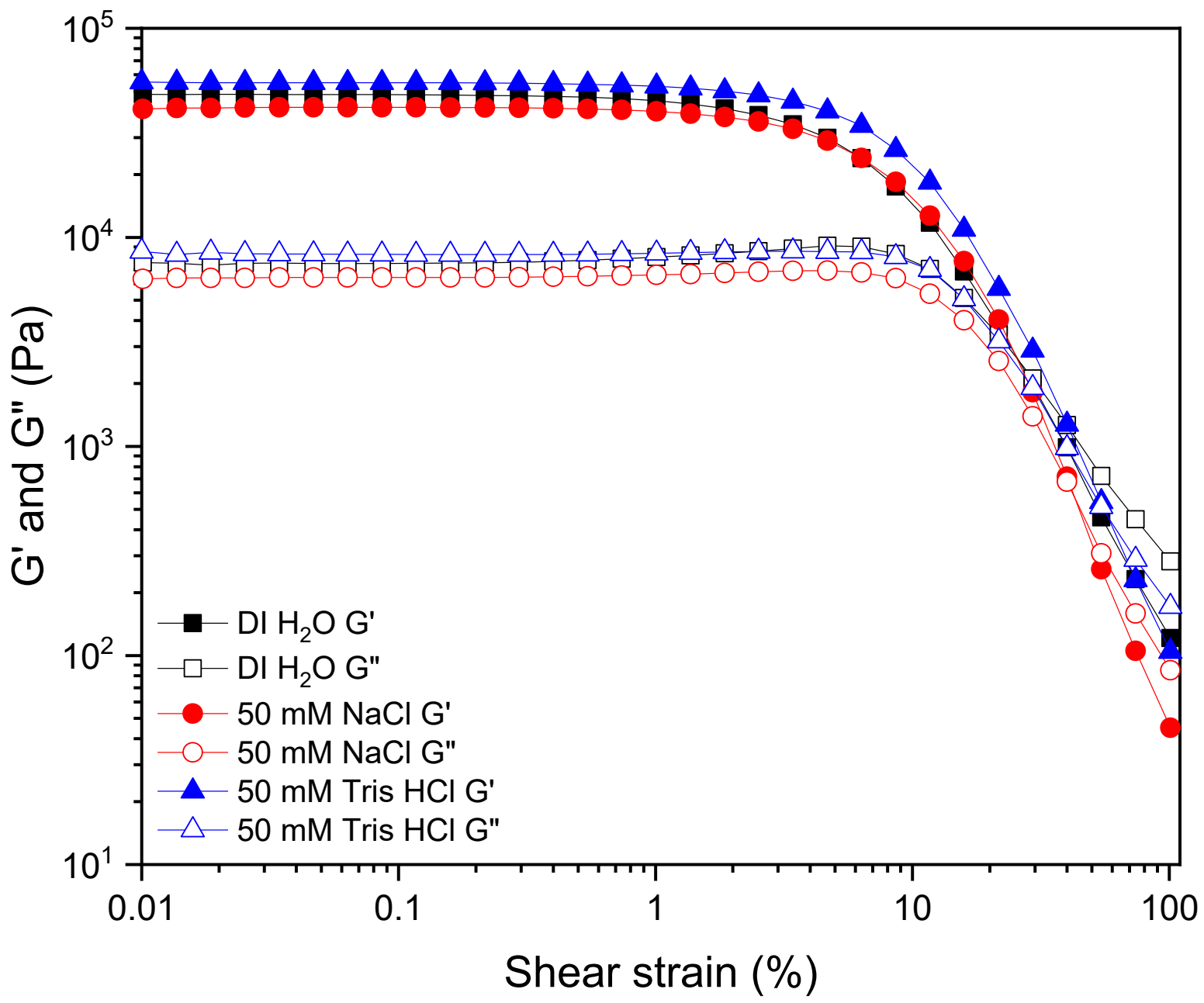

Figure S5. Amplitude sweeps of P*t*BuSMA-*b*-PNAM BCP 1:1 gels at fixed BCP and Ca^{2+} concentrations (15% w/v and Z = 1.57), prepared in different aqueous media, namely deionised water, NaCl (50 mM) and Tris HCl buffer (50 mM) solutions. The pH was 7.41, 7.60 and 7.64 for the DI H_2O, NaCl and Tris HCl buffer samples, respectively. The comparable elastic and viscous moduli of these BCP gels suggest that the effect of decreasing ionic strength (DI H_2O), or increasing concentration of monovalent cations (NaCl), on gelation is negligible.

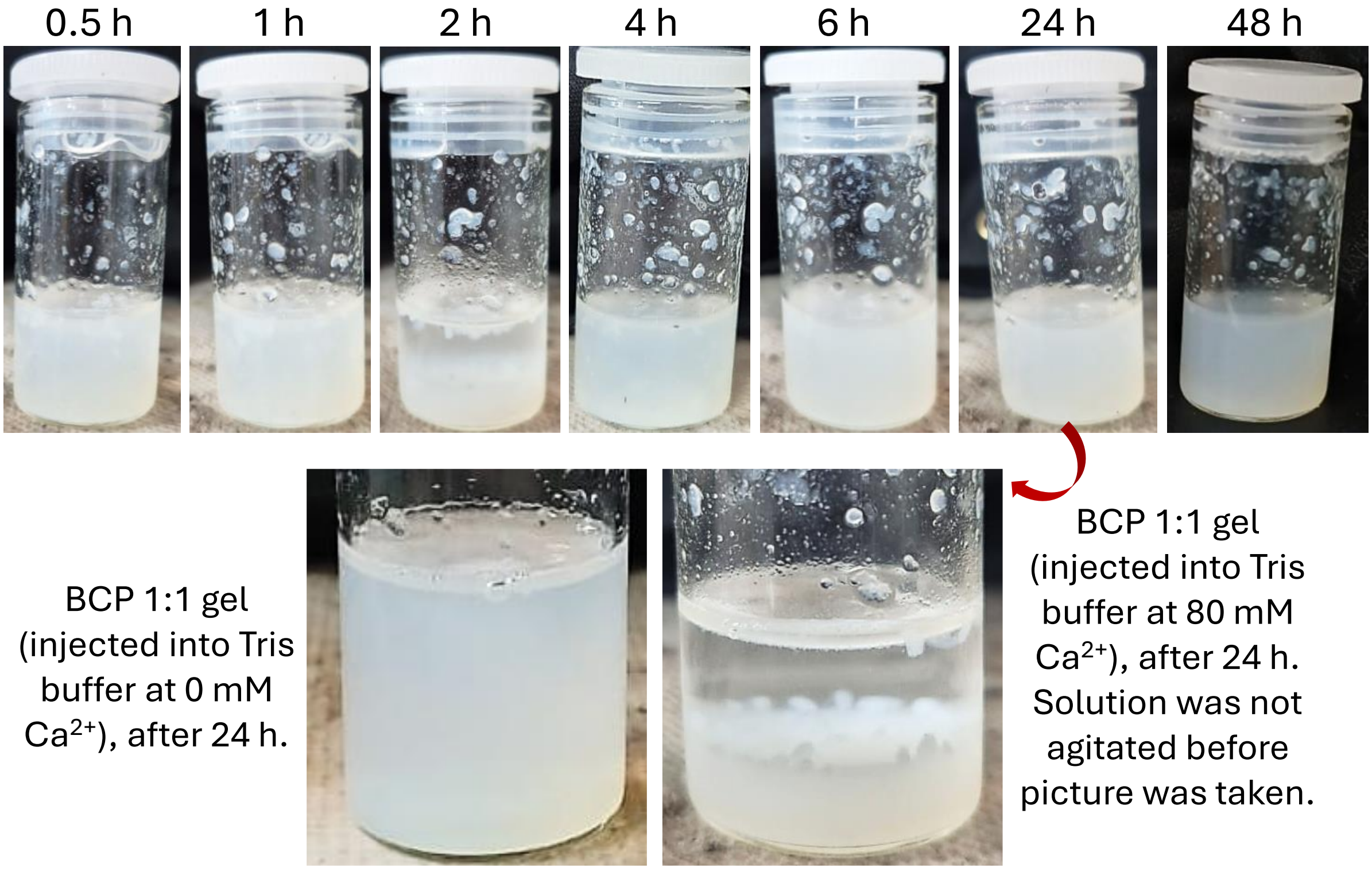

Figure S6. A representative BCP 1:1 gel (7.5% w/v, Z = 1.57 or $[Ca^{2+}]$ = 80 mM, 1 mL) was injected into Tris HCl buffer (5 mL, 80 mM Ca^{2+}) at room temperature, in order to assess if the presence of a similar calcium concentration in the injection medium would affect the gel's dissolution kinetics. The dissolution of the gel was tracked visually, whereby the solution was agitated at specified time intervals and photographed. For gels injected into Tris HCl buffer (0 mM calcium), nearly all the gel had dissolved, yielding an opaque solution (**Figure 9d** in the main text). However, gels injected into Tris buffer (80 mM calcium) dissolved much slower and could still be observed as globules suspended in solution (which sedimented if left undisturbed) after 24 h.
